# Suppression of RNA-dependent RNA polymerase 6 in tomatoes allows potato spindle tuber viroid to invade basal part but not apical part including pluripotent stem cells of shoot apical meristem

**DOI:** 10.1371/journal.pone.0236481

**Published:** 2020-07-27

**Authors:** Takashi Naoi, Syoya Kitabayashi, Atsushi Kasai, Kohei Sugawara, Charith Raj Adkar-Purushothama, Mineo Senda, Tatsuji Hataya, Teruo Sano

**Affiliations:** 1 Graduate School of Agriculture, Hokkaido University, Sapporo, Japan; 2 Faculty of Agriculture and Life Science, Hirosaki University, Hirosaki, Japan; 3 Département de Biochimie, Faculté de Médecine des Sciences de la Santé, Pavillon de Recherche Appliquée au Cancer, Université de Sherbrooke, Sherbrooke, Québec, Canada; 4 Research Faculty of Agriculture, Hokkaido University, Sapporo, Japan; Instituto de Biologia Molecular y Celular de Plantas, SPAIN

## Abstract

RNA-dependent RNA polymerase 6 (RDR6) is one of the key factors in plant defense responses and suppresses virus or viroid invasion into shoot apical meristem (SAM) in *Nicotiana benthamiana*. To evaluate the role of *Solanum lycopersicum* (Sl) RDR6 upon viroid infection, SlRDR6-suppressed (SlRDR6i) ‘Moneymaker’ tomatoes were generated by RNA interference and inoculated with intermediate or lethal strain of potato spindle tuber viroid (PSTVd). Suppression of SlRDR6 did not change disease symptoms of both PSTVd strains in ‘Moneymaker’ tomatoes. Analysis of PSTVd distribution in shoot apices by *in situ* hybridization revealed that both PSTVd strains similarly invade the basal part but not apical part including pluripotent stem cells of SAM in SlRDR6i plants at a low rate unlike a previous report in *N*. *benthamiana*. In addition, unexpectedly, amount of PSTVd accumulation was apparently lower in SlRDR6i plants than in control tomatoes transformed with empty cassette in early infection especially in the lethal strain. Meanwhile, SlRDR6 suppression did not affect the seed transmission rates of PSTVd. These results indicate that RDR6 generally suppresses PSTVd invasion into SAM in plants, while suppression of RDR6 does not necessarily elevate amount of PSTVd accumulation. Additionally, our results suggest that host factors such as RDR1 other than RDR6 may also be involved in the protection of SAM including pluripotent stem cells from PSTVd invasion and effective RNA silencing causing the decrease of PSTVd accumulation during early infection in tomato plants.

## Introduction

Viroids are single-stranded (ss), circular RNA molecules known as the smallest plant pathogens, ranging from 246 to 401 nucleotides (nt) in length [1, 2]. They are non-coding RNA pathogens and replicate autonomously depending on the transcriptional machinery of the invaded host cells [3, 4]. Viroids are classified into two families, *Pospiviroidae* and *Avsunviroidae*, by the site and mode of replication, structural nature, and whether they have hammerhead ribozyme motifs [2, 5]. The members of family *Pospiviroidae* have five structural and functional domains (terminal left, TL; pathogenicity, P; central conserved, C; variable, V; and terminal right, TR) on the rod-like secondary structures and replicate in the nucleus of invaded cells through an asymmetric rolling-circle mechanism. By contrast, the members of family *Avsunviroidae* form branched secondary structures and replicate in the chloroplast of invaded cells through a symmetric rolling-circle mechanism and exhibit self-slicer activity by hammerhead ribozyme motifs contained in their own strands of both polarities.

Potato spindle tuber viroid (PSTVd) of the genus *Pospiviroid* has a wide host range and mainly infects plants of families *Solanaceae* and *Asteraceae*. Host species sensitive to PSTVd infection exhibit disease symptoms of various degrees [6]. Tomatoes (*Solanum lycopersicum*) are particularly susceptible to PSTVd, and the severity of disease symptoms varies from almost asymptomatic to lethal depending on cultivar. ‘Rutgers’, a highly susceptible tomato cultivar, is used as an assay plant for PSTVd. PSTVd is classified into four strains (mild, intermediate, severe, and lethal) according to the severity of disease symptoms appearing on ‘Rutgers’ [7]. Mild strains of PSTVd tend to have lower accumulation in an infected host than severe strains, while the severity of disease symptoms does not necessarily correlate with the accumulation of PSTVd [8–10].

Viroids are potent inducers and targets of RNA silencing, a defense mechanism that specifically cleaves foreign invaders such as viruses and transposons in a sequence-dependent manner in infected host cells, due to their highly base-paired stem-loop structures and partially double-stranded (ds) replicative intermediates formed transiently during replication [5]. When viroids proliferate and accumulate to a certain threshold in an infected plant cell, RNA silencing is induced, and viroids are processed by an RNase III-like enzyme called Dicer-like (DCL) into 21–24 nt small nucleotide fragments called viroid-specific small RNA (vd-sRNA). The vd-sRNA of pospiviroids is 21–24 nt, while those of avsunviroids are processed into 21 or 22 nt lengths [11–13]. Vd-sRNAs are recruited by RNA-induced silencing complex (RISC) including Argonaute (AGO), which is an RNase H-like enzyme. Guided by its incorporated vd-sRNA, RISC directs the cleavage of complementary target viroid RNA. The vd-sRNAs in RISC also suppress the expression of host genes with complementary sequences to the vd-sRNAs and are involved in disease-related symptom expression of the viroids [14–16]. These findings suggest the close relationship between RNA silencing and disease symptom expression of viroids. In general, RNA silencing is maintained and even amplified by RNA-dependent RNA polymerases (RDRs). Namely, RDRs convert RNAs with aberrant features into dsRNAs, which are then processed again by DCLs into 21–24 nt small RNAs called secondary small interfering (si) RNAs. This RDR-mediated phase to produce secondary siRNA plays an important role to amplify the effect of RNA silencing [17]; however, this process has not yet been clarified in the case of viroid infection.

Six RDRs exist in *Arabidopsis thaliana*, and RDR1 and RDR6 are involved in the defensive response against plant viruses [17–19]. RDR1 influences susceptibility to tobacco mosaic virus (TMV) and potato virus X (PVX) in *A*. *thaliana* [20]. *Nicotiana benthamiana* is a natural loss-of-function mutant of *N*. *benthamiana* (Nb) RDR1 [21], and transgenic *N*. *benthamiana* expressing *Medicago truncatula* (Mt) RDR1 suppressed development of lethal disease symptoms and precluded invasion of TMV into the shoot apical meristem (SAM) [22]. RDR6 is implicated in the defense against cucumber mosaic virus (CMV) in *A*. *thaliana* [23, 24]. The role of RDR6 in virus-infected plants was tested with down regulation of RDR6 in *N*. *benthamiana* (NbRDR6i plant) [25]. Suppression of RDR6 in *N*. *benthamiana* by RNA interference (RNAi) changes plants hypersusceptible to PVX, potato virus Y (PVY), and CMV in combination with the Y satellite, but not TMV, tobacco rattle virus (TRV), turnip crinkle virus (TCV), or CMV alone. Furthermore, RDR6 is required not for generation or movement of the silencing signal but rather for synthesis of dsRNA precursors to produce the secondary siRNAs, which play a role in antiviral silencing and preclude the invasion of PVX into the SAM. Conversely, despite a high accumulation of virus-derived siRNA, efficiency of PVX- and/or plum pox virus (PPV)-driven virus-induced gene silencing (VIGS) and RNA-directed DNA methylation are suppressed in NbRDR6i plants [26]. In another transgenic *N*. *benthamiana* line expressing dsRNA of RDR6 [27], suppression of RDR6 in *N*. *benthamiana* increased host susceptibility to TCV, PVX, and TMV and promoted the invasion of TMV into shoot apices. Besides positive-sense ssRNA viruses, the susceptibility to infections of rice stripe virus (RSV) possessing negative-sense ssRNA genome and rice dwarf virus (RDV) possessing dsRNA genome was increased in *Oryza sativa* (Os) RDR6-suppressed rice plants [28, 29]. In the case of viroid, RDR1 expression was enhanced in PSTVd-infected ‘Rutgers’ tomatoes and hop stunt viroid (HSVd)-infected ‘Suyo’ cucumbers, suggesting the involvement of RDR1 in the anti-viroid defense [30, 31]. The role of RDR6 in plants infected with viroid was first analyzed using NbRDR6i plants infected with HSVd [32]. The NbRDR6i plant was used as scion and grafted on a transgenic *N*. *benthamiana* expressing dimeric forms of HSVd. Thus, HSVd-induced symptom expression was suppressed on NbRDR6i scion, while the accumulation of HSVd was similar to that in symptomatic wild-type scions or in HSVd-transgenic rootstock. In another host-viroid combination (i.e, *N*. *benthamiana* and PSTVd), the accumulation of PSTVd in the early infection stage was increased in NbRDR6i plants as compared with that of control plants [33]. Similarly, the role of RDR6 in the accumulation of PSTVd in the non-transgenic, wild-type *N*. *benthamiana* plant was examined by VIGS technique [34]. The VIGS-suppression of RDR6 in *N*. *benthamiana* plant increased the accumulation of both PSTVd genome and small RNAs derived from PSTVd, which was consistent with the previous finding by Di Serio *et al*. [33]. Additionally, suppression of RDR6 in *N*. *benthamiana* did not affect the severity or the expression of disease symptoms of PSTVd [33]. These findings indicate that RDR6 of *N*. *benthamiana* has a function to suppress PSTVd accumulation.

Plants must protect their SAM which has the ability to develop into organs including various tissues and reproductive organs, from the invasion of viruses and viroids. SAM includes pluripotent stem cells which repeatedly divide, and continuously generate two type cells that maintain pluripotency of stem cells or that differentiate into the all above-ground organs and tissues of plants [35–40]. It was suggested in previous reports that an RNA surveillance system involving post-transcriptional gene silencing (PTGS) suppresses the invasion of RNA viruses into SAM, and CMV was excluded from SAM of *N*. *benthamiana* along with the accumulation of CMV-derived small RNA [41, 42]. In the tomato and *N*. *benthamiana*, PSTVd invaded vascular systems and other tissues but not the SAM, suggesting that the plant surveillance system prevents PSTVd from invading apical meristems [43, 44]. RNAi-mediated suppression of RDR6 in *N*. *benthamiana* allowed PVX and PSTVd to invade into SAM [25, 33]. These findings suggest that RDR6 of tomatoes is also involved in the surveillance system that is able to suppress PSTVd entry into SAM.

The invasion of virus and viroid into the SAM is often associated with disease symptoms, as in the case of SAM invasion of PVX in NbRDR6i plants, for example [25]. Peach latent mosaic viroid (PLMVd) most likely interferes with an early step of chloroplast development in SAM resulting in albino-variegated phenotypes resembling those of certain peach (*Prunus persica*) mutants in which maturation of rRNA in plastid is impaired [45, 46]. Alternatively, chrysanthemum stunt viroid (CSVd) induced disease symptoms in two *Argyranthemum* cultivars regardless of invasion into SAM, and invasion of PSTVd into SAM of NbRDR6i plants did not change the severity of disease symptoms [47, 33]. These reports suggest that the correlation between virus/viroid entry into SAM and presence of disease symptoms may differ depending on the combination of virus/viroid and host plant.

As previously described, RDR6, one of the key factors in the RNA silencing mechanism, plays an important role in plant anti-viroid defense responses in the model plant *N*. *benthamiana*. In this study, we created an *S*. *lycopersicum* (Sl) RDR6-suppressed transgenic tomato plant and analyzed the effects of SlRDR6 suppression on PSTVd pathogenicity, SAM invasion, accumulation, and seed transmission. The results of this study will provide new insights into the roles of RDR6 in defense response against viroid in tomato plants.

## Materials and methods

### Generation of SlRDR6-suppressed transgenic tomato line

A transgenic ‘Moneymaker’ tomato line in which SlRDR6 expression was suppressed by RNAi was generated as previously described [48]. The artificial gene SlartRDR6 was constructed based on the sequence of tomato homolog genes (SlRDR6a; Solyc04g014870 and SlRDR6b; Solyc08g075820) of *A*. *thaliana* RDR6 registered in the tomato genome database (https://solgenomics.net/organism/Solanum_lycopersicum/genome) (S1 Fig). In the inverted-repeat (IR) SlartRDR6 construct, a set of the SlartRDR6 sequence was placed in the head-to-head direction across an intron sequence to create an IR sequence (S1 Fig). The binary vector pIG121-Hm containing the IR-SlartRDR6 construct downstream of the CaMV-35S promoter was introduced into *Agrobacterium tumefaciens* strain EHA105 to transform the ‘Moneymaker’ tomato. A T3-generation of SlRDR6-suppressed tomato lines was selected by repeated kanamycin selection and self-fertilization, and line 91B was used as the SlRDR6i plant in PSTVd infection assays. As a control, the transgenic tomato line empty cassette (EC) transformed with pIG121-Hm containing an empty cassette from our previous study [48] was also used in this work.

### Detection of the CaMV-35S promoter sequence by PCR

Total nucleic acid was extracted from leaves of healthy transgenic tomato plants by the cetyltrimethylammonium bromide (CTAB) method [49] and used as a template of polymerase chain reaction (PCR) to amplify a part of the CaMV-35S promoter sequence and the actin gene. The PCR products were fractionated in 7.5% polyacrylamide gel containing 1×Tris-acetate-EDTA (TAE) buffer. PCR was performed with One Taq DNA polymerase (New England BioLabs) according to the manufacturer’s protocol. The primer sets used for PCR are described in S1 Table.

### Preparation of DIG-labeled cRNA probes for Southern-blot or Northern-blot hybridization

DIG-labeled cRNA probes used for Southern-blot or Northern-blot hybridization were transcribed from plasmid constructs including a full length SlartRDR6 sequence or a partial sequence of CaMV-35S promoter or a dimer of minus strand PSTVd-Dahlia (S1 and S2 Figs) [50]. The plasmid construct was digested with restriction enzymes and treated with phenol:chloroform (1:1, vol/vol). The ethanol precipitate was air-dried, suspended in ultrapure water, and used for *in vitro* transcription with T3 or T7 RNA polymerase and DIG RNA labeling mixture (Roche Diagnostics). The transcripts were collected by ethanol precipitation using LiCl, air-dried, and suspended in ultrapure water. Concentration and size of the probe was confirmed by agarose gel electrophoresis.

### Preparation of genomic DNA and analysis of the copy number of transgenes by Southern-blot hybridization

Total nucleic acid was treated with DNase-free RNaseA (Nippon Gene) to obtain RNA-free genomic DNA. Genomic DNA samples were digested with *Eco*RI or *Bam*HI (Thermo Fisher Scientific), electrophoresed at 50 V (4 V/cm) for 8 h in a 1.0% agarose gel (1× TAE buffer), transferred to a nylon membrane (Biodyne plus) after NaOH-denaturation followed by HCl-neutralization, and hybridized with DIG-labeled cRNA probe for the CaMV-35S promoter sequence (S2 Fig). Hybridized signals were visualized using the Chemidoc-XRS imaging system (Bio-Rad Laboratories).

### Preparation of PSTVd inoculum and mechanical inoculation

Low molecular weight (LMW) RNA containing PSTVd was extracted from ‘Rutgers’ tomato plants inoculated with dimeric transcripts of PSTVd-Intermediate (-Int) (Accession No. M16826) or PSTVd-RG1 (Accession No. U23058) with the method described in Sano *et al*. [51]. Inoculums were made from the LMW RNA samples containing approximately equal amounts of different types of PSTVd: PSTVd-Int was dissolved at a concentration of 100 ng LMW RNA/μL and PSTVd-RG1 at 300 ng LMW RNA/μL in a 50 mM sodium phosphate buffer (pH 7.5) containing 1 mg/mL bentonite. The relative concentration of PSTVd in each LMW RNA sample was estimated by comparing the signal intensity of Northern-blot hybridization. For mechanical inoculation, an aliquot (10 μL) was placed on cotyledons of transgenic tomato seedlings dusted with carborundum (600 mesh) and gently rubbed 10 times against the leaf using a sterile glass bar. Each PSTVd was inoculated onto 15 seedlings of transgenic plants. After inoculation, plants were incubated in a culture room at 22°C (night)–25°C (day), 16 h day-length with fluorescent light (3000–4000 lux).

### Sampling of leaf disks and total RNA extraction

To detect SlartRDR6 transgene-derived transcripts (SlartRDR6-transcripts) and transgene-derived small RNA (SlartRDR6-sRNA), a total of 10 leaf disks (about 10 mm in diameter) were collected from 10 individual healthy transgenic tomato plants for RNA extraction.

Leaves were sampled from PSTVd-infected transgenic tomato plants at 5, 10, 15, 20, and 25 days post inoculation (dpi). PSTVd-inoculated plants were divided into three groups (biological replicate), each of which consists of five individual plants, and a total of five leaf disks (one leaf disk per individual plant) collected from each group were pooled for total RNA extraction (S3 Fig).

Total RNA was extracted using Trizol Reagent (Thermo Fisher Scientific) according to manufacturer’s protocol. Total RNA samples were used for RT-qPCR and Northern-blot hybridization.

### Fractionation of low molecular weight RNA

Furthermore, to analyze small transgene (SlartRDR6-sRNA) or viroid (PSTVd-sRNA) RNAs, LMW RNA was prepared from the aforementioned total RNA samples. An equal volume of 4 M LiCl was added to samples containing 20–30 ng of total RNA for fractionation.

The LMW RNA fraction soluble in 2 M LiCl was treated with RNase-free DNase I (RQ1 DNase; Promega), dissolved in 30 μL of distilled water and used for Northern-blot hybridization analyses.

### Detection of RNAs by Northern-blot hybridization

To detect PSTVd genomic RNA and SlartRDR6-transcripts by Northern-blot hybridization, total RNA samples were denatured at 65°C for 10 min in the presence of formamide and formaldehyde. Denatured total RNA samples were electrophoresed in 1.2% (w/v) agarose gel containing formaldehyde at a final concentration 0.66 M or 2.2 M in 1× 3-(N-morpholino)propanesulfonic acid (MOPS) buffer. Fractionated RNA in agarose gel was transferred to a nylon membrane (Biodyne plus) by vacuum blotting for 20 min (5 mm Hg) and hybridized with a DIG-labeled cRNA probe for PSTVd [51] or SlartRDR6 (S1 Fig).

To detect PSTVd-sRNA and SlartRDR6-sRNA by Northern-blot hybridization, LMW RNA samples were denatured at 68°C for 10 min in a sample buffer containing 50% urea and electrophoresed in 12% polyacrylamide gel (acrylamide:bisacrylamide = 19:1, containing urea at a final concentration 8 M) in 1×Tris-borate-EDTA buffer. Fractionated RNA in polyacrylamide gels was transferred to a nylon membrane (Biodyne plus) by contact blotting at 25°C overnight and hybridized with a DIG-labeled cRNA probe for PSTVd or SlartRDR6.

Hybridized signals were visualized using the Chemidoc-XRS imaging system (Bio-Rad Laboratories) and quantified using Quantity One (version 4.6.2) software package.

### Detection of PSTVd genomic RNA and endogenous mRNAs by RT-qPCR

Total RNA (5 μg) extracted using Trizol Reagent (Thermo Fisher Scientific) was treated with RNase-free DNase I (RQ1 DNase; Promega) and used for reverse transcription. cDNA was synthesized from 500 ng of the RNA using random hexamer primers and Superscript IV VILO Master Mix (Invitrogen). qPCR analysis was performed using Brilliant III Ultra-Fast SYBR Green QPCR Master Mix (Agilent Technologies) and AriaMx Real-Time PCR G8830A (Agilent Technologies). The PCR primers for PSTVd genomic RNA and endogenous *SlRDR1* and *SlRDR6* mRNAs were made according to previous reports [15, 52], and their sequences are described in S1 Table. The RT-qPCR results were normalized to the β-actin gene, and relative accumulation or transcript levels were calculated using the 2^−ΔΔC(t)^ method [53].

### Detection of PSTVd in shoot apices by *in situ* hybridization

Shoot apices (about 3–5 mm from the top) of PSTVd-infected transgenic tomato plants were collected at 30–35 dpi for paraffin embedding. Collected shoot apices were fixed with paraformaldehyde and glutaraldehyde, dehydrated, and embedded in paraffin. The embedded samples were cut in longitudinal sections (approximately 10 μm thick) and placed on MAS-GP glass slides (Matsunami Glass). Hybridization solution containing 60% formaldehyde and DIG-labeled cRNA probe for PSTVd was dropped onto glass slides, and hybridization was performed at 48°C for 36–40 h. After four washings at 50°C and blocking treatment for 30 min, the sections on glass slides were incubated with blocking solution containing alkaline phosphatase-conjugated anti-DIG antibody. After four further washings, alkaline phosphatase was detected by colorimetric reaction based on hydrolysis of 5-bromo-4-chloro-3-indolyl phosphate and reduction of nitroblue tetrazolium in the dark. Blue-violet staining indicating a presence of PSTVd was observed with the upright microscope (DM2500 LED; Leica).

### Analysis of seed transmission rate in PSTVd-infected tomatoes by Northern-blot hybridization

Some of the PSTVd-infected EC and SlRDR6i plants were replanted and grown to obtain fruits. Seeds obtained from each fruit were surface-sterilized by dipping for 1 min in 1% effective hypochlorous acid solution. About 20 seeds were sown per fruit, and each seedling was used for nucleic acid extraction by the CTAB method. Each nucleic acid sample was used for Northern-blot hybridization to check PSTVd infection in the seeds.

## Results

### Characterization of an SlRDR6-suppressed transgenic tomato

To evaluate the level of SlRDR6 suppression in the selected transgenic ‘Moneymaker’ tomato line 91B, the copy number and expression level of the SlartRDR6 transgene and the accumulation of transgene-derived small RNA were analyzed.

The presence of the transgene was analyzed by PCR amplification of a part of the 35S promoter sequence and was positive in line 91B (S4A Fig). The copy number of transgenes was assayed by Southern-blot hybridization using a DIG-labeled cRNA probe for the CaMV-35S promoter sequence. In transgenic tomato line 91B, a single band was detected in both *Eco*RI and *Bam*HI digestions, suggesting that this line contains a single copy of the transgene in the genomic DNA (S4B Fig).

**Fig 1:**
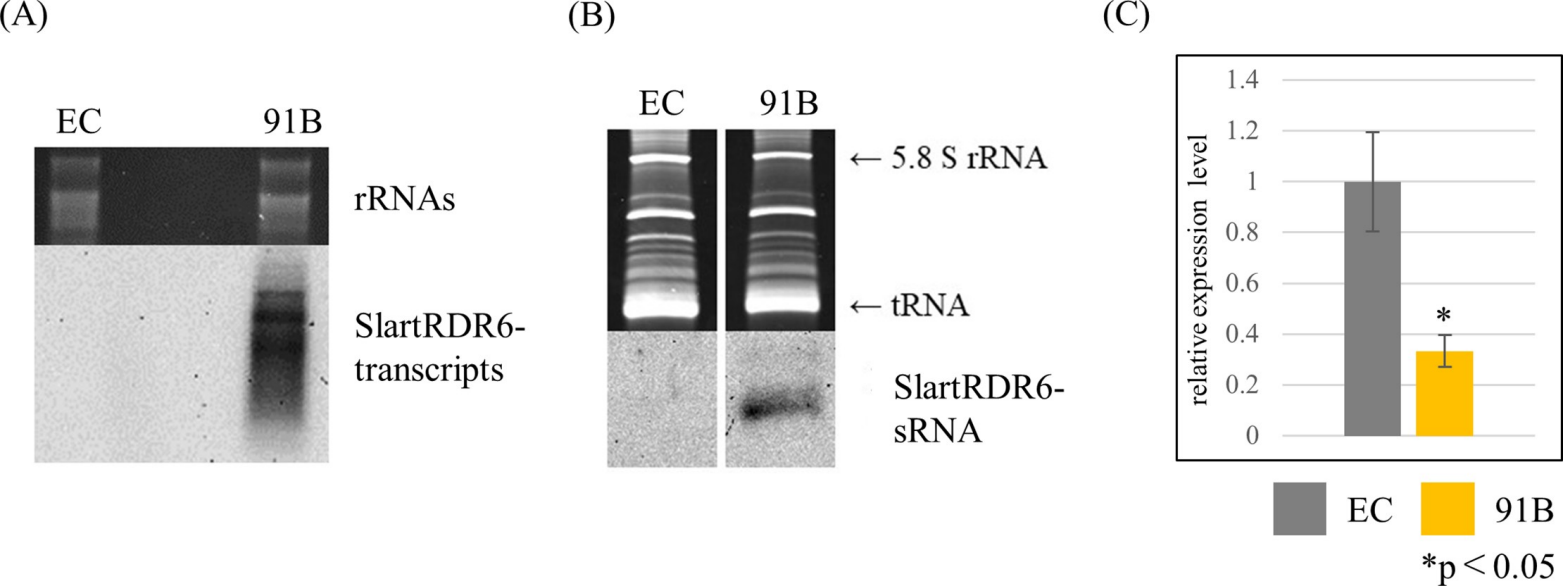
Evaluation of SlRDR6 suppression efficiency. Transgenic tomato plants were generated to analyze a role of SlRDR6 upon PSTVd infection. (A) SlartRDR6-transcripts and (B) SlartRDR6-sRNA were detected by Northern-blot hybridization with DIG-labeled cRNA probe for SlartRDR6. rRNAs and tRNA were stained with ethidium bromide and used as a loading control. SlartRDR6-transcripts and SlartRDR6-sRNA were only detected in line 91B. (C) Expression levels of endogenous *SlRDR6* mRNA were analyzed by RT-qPCR performed with the PCR primers for the endogenous *SlRDR6* gene. Mean values are based on three biological replicates of the pooled sample which is collected from five individual plants. The relative expression levels were calculated with the value of EC plants as a standard. The expression level of endogenous *SlRDR6* mRNA in line 91B was significantly reduced to approximately 40% of that in EC plants. The significant decrease of *SlRDR6* expression level in line 91B was confirmed by Welch’s *t*-test.

Transcripts from the IR-SlartRDR6 transgene (SlartRDR6-transcripts) and transcript-derived small RNA (SlartRDR6-sRNA) were detected by Northern-blot hybridization using a DIG-labeled cRNA probe for SlartRDR6. It was confirmed that both SlartRDR6-transcripts and SlartRDR6-sRNA accumulated at high levels in line 91B (Fig 1A and 1B), indicating that the transgene was expressed strongly and induced RNA silencing efficiently in line 91B.

Therefore, we analyzed the expression level of endogenous *SlRDR6* mRNA in line 91B by RT-qPCR. As expected, the expression level of *SlRDR6* in line 91B was decreased to approximately 40% of that in EC plants (Fig 1C).

**Fig 2:**
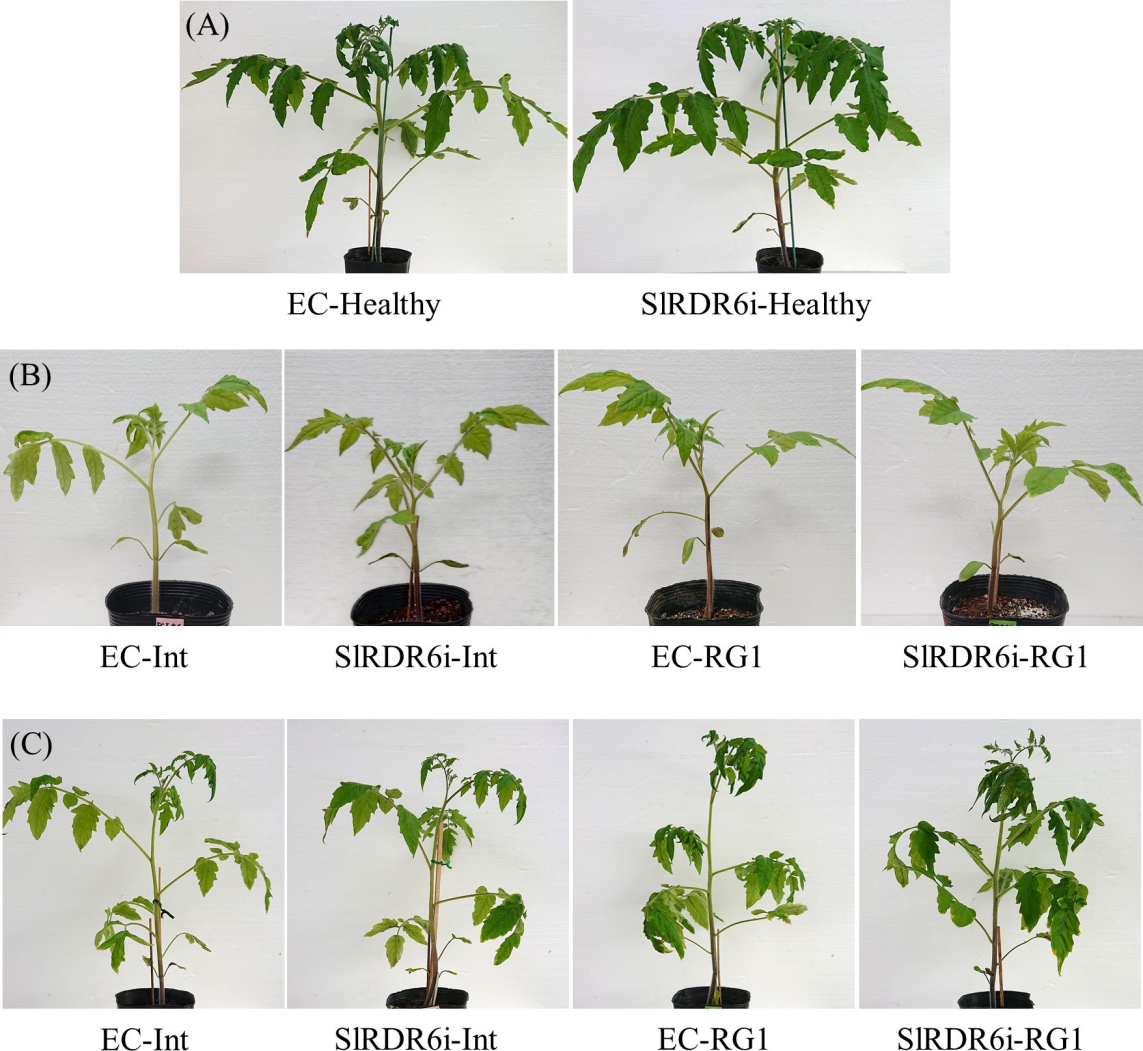
PSTVd-infected transgenic ‘Moneymaker’ tomato plants. PSTVd-Int or PSTVd-RG1 was inoculated to EC and SlRDR6i plants. (A) Phenotypes of transgenic ‘Moneymaker’ tomato plants. At 30 dpi, no clear difference of phenotypes was observed between healthy transgenic plants. (B) At 15 dpi, both PSTVd-infected transgenic tomato plants were asymptomatic. (C) At 30 dpi, the disease symptoms of PSTVd-Int or PSTVd-RG1 reached the same intensity between EC and SlRDR6i plants.

Because 91B plants was confirmed to be SlRDR6-suppressed plants as expected (line 91B is hereinafter referred to SlRDR6i plant), we examined the growth of SlRDR6i plants under our experimental conditions before starting the PSTVd infection assay. SlRDR6i plants did not show a significant difference in growth or phenotypic appearance compared with EC plants (Fig 2A); however, productivity of fruit was apparently poor.

### SlRDR6 suppression does not impair the tolerance of ‘Moneymaker’ tomatoes to PSTVd infection

To investigate a role of SlRDR6 in defense responses against viroid infection, two different PSTVd strains, PSTVd-Int (intermediate strain) and PSTVd-RG1 (lethal strain), were used for infection assays. PSTVd-RG1 is known to induce lethal symptoms that are more severe than PSTVd-Int in ‘Rutgers’ tomatoes [54]. Because ‘Moneymaker’ tomatoes are tolerant to PSTVd [55, 56], both EC and SlRDR6i plants were asymptomatic even during infection with lethal PSTVd-RG1 isolate until 15 dpi (Fig 2B). Subsequently, both EC and SlRDR6i plants developed moderate leaf curling at 30 dpi with PSTVd-Int, while severe leaf curling with vein necrosis occurred with PSTVd-RG1 (Fig 2C). By contrast, regardless of the PSTVd strains used for infection, no substantial differences could be seen in the severity of symptoms between EC and SlRDR6i plants (Fig 2C), indicating that ‘Moneymaker’ tomatoes do not lose its tolerance to PSTVd during SlRDR6 suppression.

### SlRDR6 suppression allowed PSTVd to invade basal part but not apical part of shoot apical meristems regardless of PSTVd strain

SAM is the tissue that gives rise to various organs in plants; hence, it is important to protect it from invasion of pathogens such as viruses and viroids. It was reported that PSTVd was not present in the SAM of *N*. *benthamiana* and tomato plants infected with PSTVd [43, 44]. Meanwhile, RDR6 was reported to suppress virus and viroid invasion into the SAM in *N*. *benthamiana*, suggesting that RDR6 plays an important role in protecting SAM from pathogens [25, 33].

**Fig 3:**
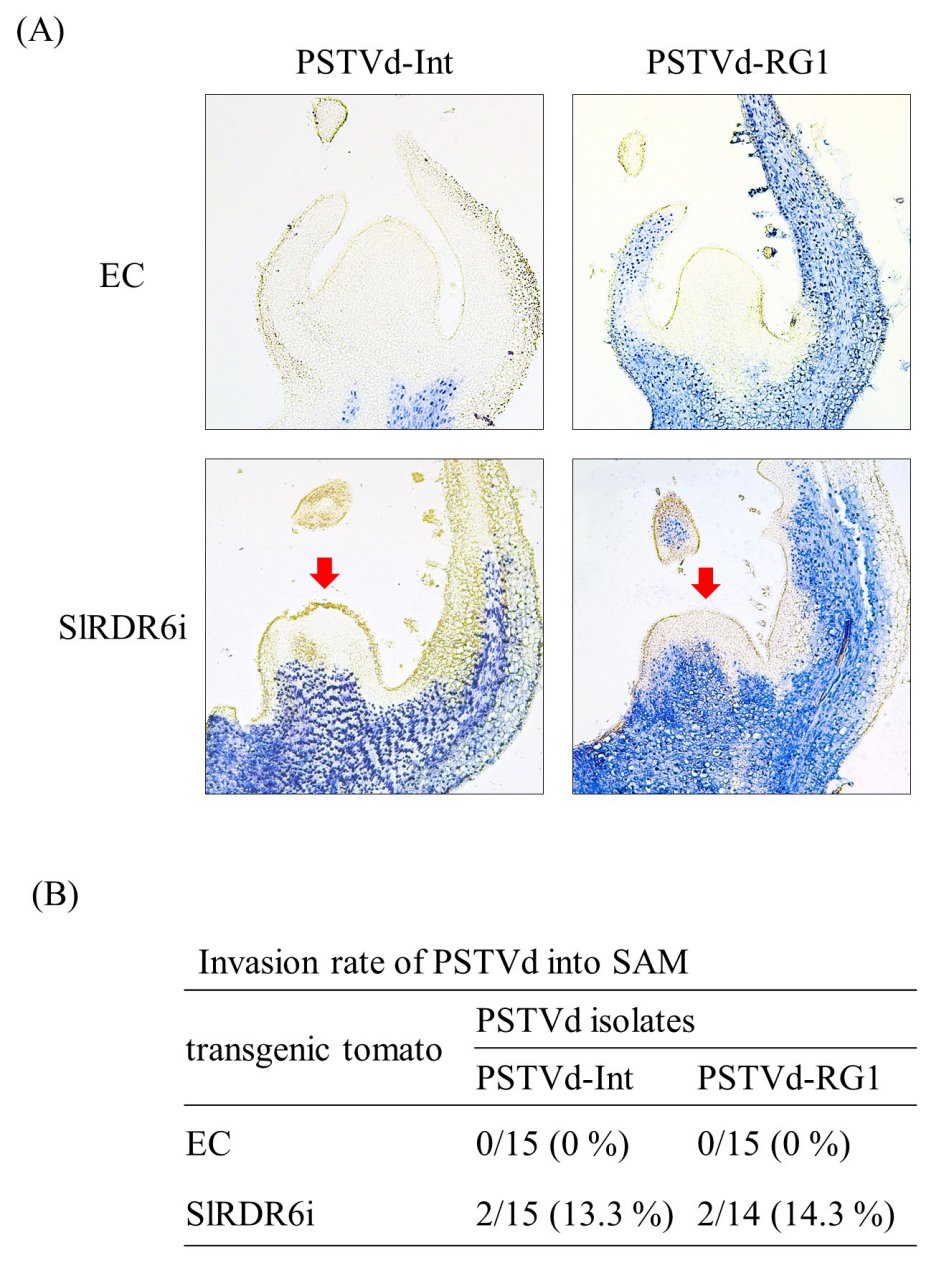
SlRDR6-suppression allowed PSTVd to invade basal part but not apical part of SAM. (A) Presence of PSTVd in shoot apices of transgenic ‘Moneymaker’ tomato plants was analyzed by *in situ* hybridization with DIG-labeled cRNA probe for PSTVd at 30–35 dpi. The blue-violet signal in the longitudinal section of shoot apices indicates the presence of PSTVd. PSTVd-Int and PSTVd-RG1 were detected in the basal part but not apical part of SAM in SlRDR6i plants (indicated with a red arrow). (B) Rates of PSTVd invasion into the SAM of EC and SlRDR6i plants. Shoot apices (14 or 15) in each test section were used in the detection of PSTVd. The invasion rates of PSTVd-Int and PSTVd-RG1 into the SAM of SlRDR6i plants were similar.

Therefore, to investigate whether SlRDR6 protects SAM from viroid invasion in tomato plants, we analyzed the distribution of PSTVd in shoot apices of the PSTVd-infected SlRDR6i ‘Moneymaker’ tomato plants by *in situ* hybridization compared with EC plants. *In situ* hybridization of longitudinal sections revealed that neither PSTVd-Int nor PSTVd-RG1 isolates invaded the SAM of EC plants at all, but they invaded the basal part of SAM in SlRDR6i plants at a rate of 13.3% and 14.3%, respectively; however, both PSTVd isolates did not invade the apical part including pluripotent stem cells of SAM (Fig 3).

### Suppression of SlRDR6 suppressed accumulation of PSTVd genomic RNA during early infection especially in the virulent PSTVd-RG1 isolate

Because some invasion of PSTVd into the SAM of SlRDR6-suppressed tomatoes was observed, the role of SlRDR6 in the defense response against viroid infection was also evaluated by analyzing the changes in accumulation of PSTVd genomic RNA in the plants.

**Fig 4:**
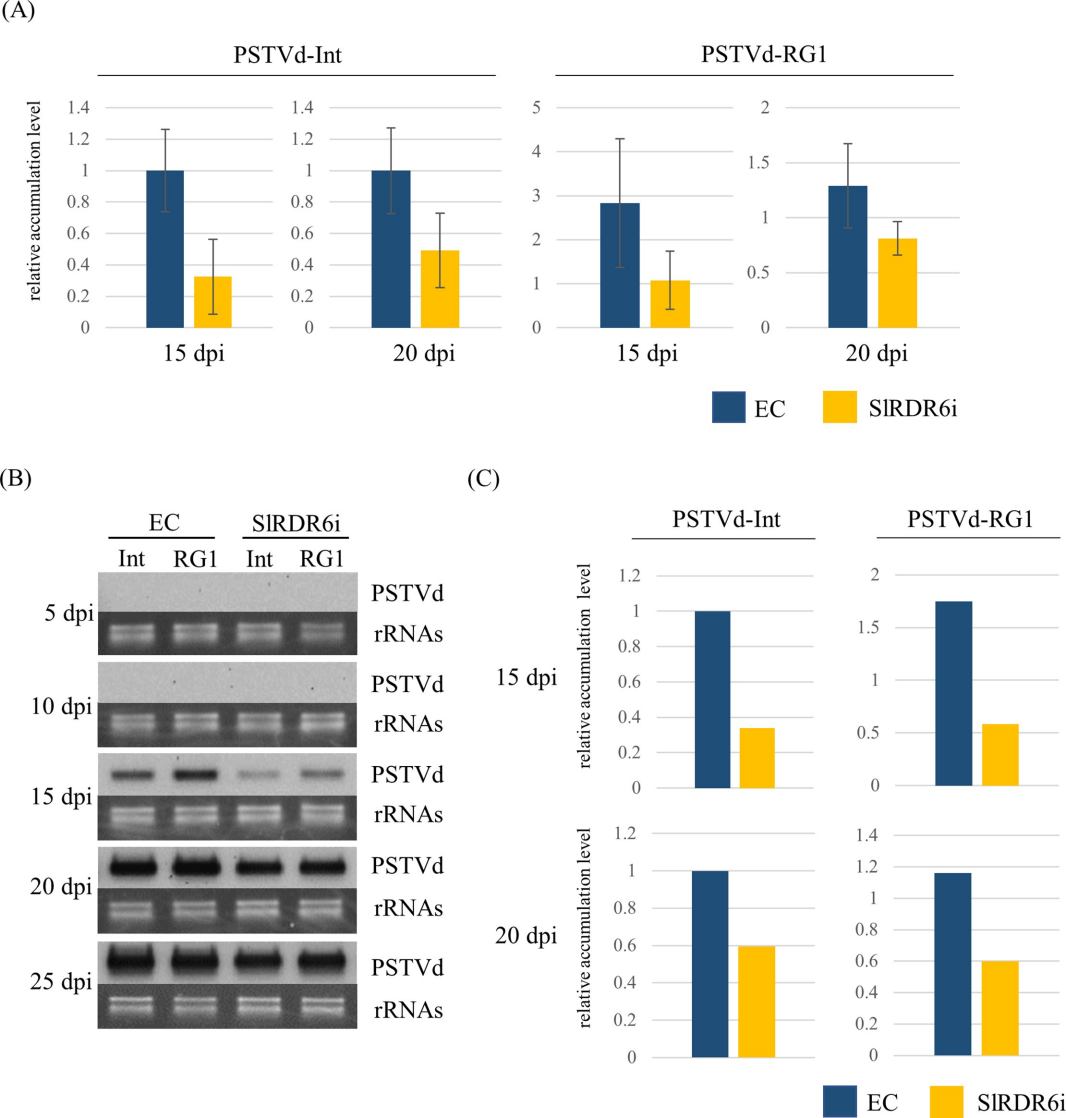
Time-course analysis of accumulation levels of PSTVd genomic RNA in transgenic ‘Moneymaker’ tomato plants. (A) Accumulation of PSTVd genomic RNA was analyzed by RT-qPCR. At 15 and 20 dpi, the accumulation levels of PSTVd-Int and PSTVd-RG1 tended to be lower in SlRDR6i than in EC plants. (B) Accumulation of PSTVd genomic RNA was analyzed by Northern-blot hybridization with DIG-labeled cRNA probe for PSTVd. rRNAs were stained with ethidium bromide and used as a loading control. At 15 and 20 dpi, the accumulation levels of PSTVd-Int and PSTVd-RG1 were lower in SlRDR6i than in EC plants. (C) Comparison of relative accumulation of PSTVd genomic RNA by Northern-blot hybridization. Signals of Northern-blot hybridization indicating the accumulation of PSTVd genomic RNA were quantified using Quantity One (version 4.6.2) software package. The relative PSTVd levels were calculated for each time point with the value of EC plants inoculated with PSTVd-Int as a standard. The accumulation of the two PSTVd strains in SlRDR6i plants at 15 dpi reached about one-third of that in EC plants. qPCR was performed with the PCR primer for PSTVd. Mean values are based on three biological replicates of the pooled sample which is collected from five individual plants.

Accumulation of PSTVd genomic RNA was first analyzed by RT-qPCR with pooled three total RNA samples because the level of PSTVd accumulation in systemic leaves was assumed to be greatly different between individual plants (S3 Fig). The accumulation of two PSTVd strains was undetectable at 10 dpi even by highly sensitive RT-qPCR. Subsequently, the accumulation of PSTVd genomic RNA tended to be lower in SlRDR6i than in EC plants at 15 dpi although no statistically significant difference of PSTVd accumulation could be observed between EC and SlRDR6i plants, because PSTVd accumulation levels were highly different between samples even which they were pooled (Fig 4A). Therefore, the equal amount of total RNA from three pooled samples was mixed and used for Northern-blot hybridization (S3 Fig).

Northern-blot hybridization revealed that the intensity of positive signals of the two PSTVd isolates Int and RG1 was lower in SlRDR6i than in EC plants at 15–20 dpi in agreement with the result of RT-qPCR assay but increased to an almost indistinguishable level at 25 dpi (Fig 4B). These hybridization signals at 15 and 20 dpi were quantified by Quantity One software (Bio-Rad Laboratories), normalized using a loading control, and used to compare the relative accumulation levels of two PSTVd strains in EC and SlRDR6i plants. Comparison of signal intensity showed that the accumulation of the two PSTVd strains in SlRDR6i plants at 15 dpi reached about one-third of that in EC plants (Fig 4C).

Because PSTVd accumulation early in the infection appeared suppressed in transgenic SlRDR6i ‘Moneymaker’ tomato plants, a second set of infection assays was conducted, and the same analyses were repeated. In this experiment, PSTVd-RG1 accumulation was again suppressed in SlRDR6i plants at 10–20 dpi (S7 Fig); however, PSTVd-Int reached detectable levels earlier at 10 dpi and accumulated slightly faster in SlRDR6i than in EC plants. Afterwards, at 15 dpi, the accumulation was reversed between EC and SlRDR6i plants (S7C Fig), although the difference was not statistically significant. These results confirmed that at least in the virulent PSTVd-RG1 isolate, the accumulation was suppressed in the transgenic SlRDR6i ‘Moneymaker’ tomatoes during early infection. However, suppression in PSTVd accumulation was limited and not stable in case of the less virulent PSTVd-Int isolate. Therefore, these results suggest that SlRDR6 affects the accumulation of PSTVd genomic RNA during early but not late infection regardless of PSTVd strain.

**Fig 5:**
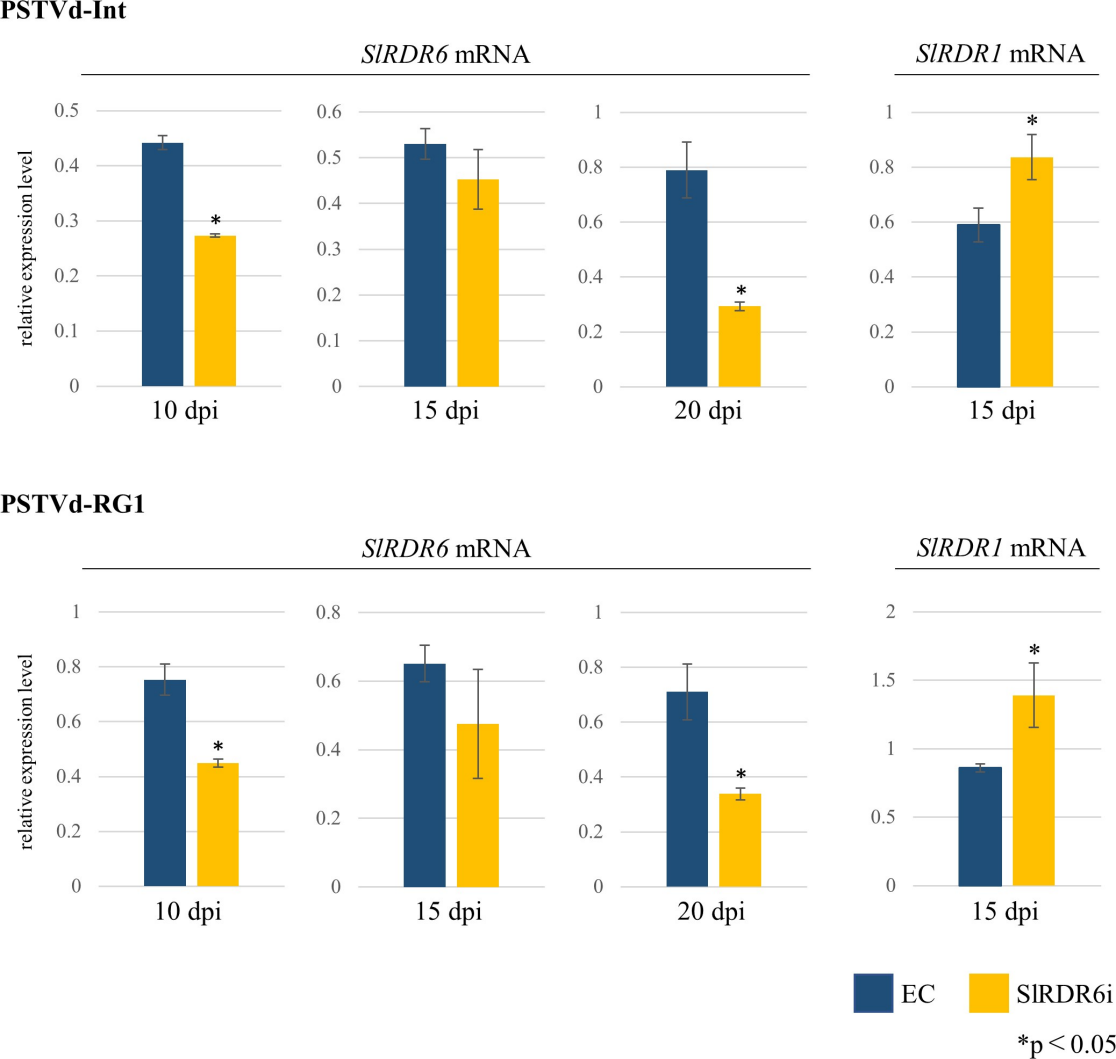
Expression levels of endogenous *SlRDR6* and *SlRDR1* mRNAs in PSTVd-infected transgenic ‘Moneymaker’ tomato plants. Expression levels of endogenous *SlRDR6* and *SlRDR1* mRNAs were analyzed by RT-qPCR. The expression levels of *SlRDR6* mRNA were significantly lower in PSTVd-infected SlRDR6i plants than in EC plants at 10 and 20 dpi except for 15 dpi. The expression levels of *SlRDR1* mRNA were significantly higher in PSTVd-infected SlRDR6i plants than in EC plants at 15 dpi. The statistically significant difference between EC and SlRDR6i plants was confirmed by Welch’s or Student’s *t*-test. The relative expression levels of endogenous *SlRDR6* and *SlRDR1* mRNAs were calculated for each time point with the value of the mock-inoculated EC plants as a standard. qPCR was performed with the PCR primer for endogenous *SlRDR6* mRNA, or endogenous *SlRDR1* mRNA. Mean values are based on three biological replicates of the pooled sample which is collected from five individual plants.

Furthermore, to investigate whether the changes in PSTVd accumulation are related to the changes in expression of the *SlRDR6* gene, Northern-blot hybridization and RT-qPCR were performed in EC and SlRDR6i plants. Because the expression level of *SlRDR6* was so low, it was only detected by RT-qPCR. The same three pooled total RNA samples used for PSTVd detection were also used for detection of endogenous *SlRDR6* mRNA. The analysis revealed that the expression of *SlRDR6* tended to decrease upon PSTVd infection (S5 Fig) and was particularly low in SlRDR6i plants compared with that of EC plants after PSTVd infection at 10 and 20 dpi except for 15 dpi (Fig 5; S5 Fig).

As well as RDR6, RDR1 is also implicated in the defense response to plant viruses, and expression of *RDR1* was enhanced in viroid-infected plants [30, 31]. Therefore, the expression level of *SlRDR1* was also analyzed by RT-qPCR. The expression of *SlRDR1* was significantly increased in SlRDR6i plants compared with EC plants at 15 dpi, at which PSTVd accumulation was lower in SlRDR6i plants than in EC plants, especially in PSTVd-RG1 infection (Fig 5; S6 Fig).

### SlRDR6 suppression does not change the relative accumulation of PSTVd-sRNA to PSTVd genomic RNA regardless of PSTVd strain

In general, RDR-mediated production of secondary siRNA plays an important role to amplify the effect of RNA silencing [17]. However, this process has not yet been clarified in the case of viroid infection, and the accumulation of PSTVd was suppressed in transgenic SlRDR6i ‘Moneymaker’ tomatoes (Fig 4; S7 Fig). Therefore, the accumulation of PSTVd-derived small RNA (PSTVd-sRNA) in SlRDR6i plants was analyzed at 15, 20, and 25 dpi.

**Fig 6:**
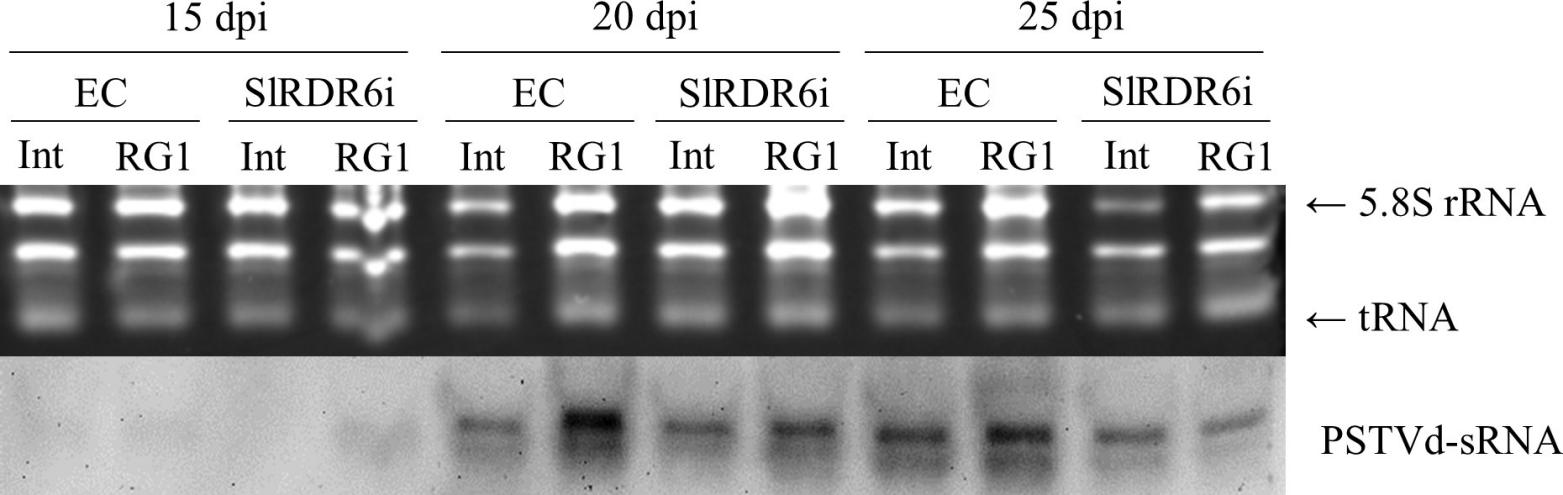
Accumulation of PSTVd-sRNA in transgenic ‘Moneymaker’ tomato plants. Accumulation of PSTVd-sRNA was analyzed by Northern-blot hybridization with DIG-labeled cRNA probe for PSTVd. Loadings were equalized by the signal intensity of rRNA and tRNA stained with ethidium bromide. PSTVd-sRNA derived from PSTVd-Int or PSTVd-RG1 genomic RNA was highly accumulated in EC plants compared with that in SlRDR6i plants.

To analyze the accumulation of PSTVd-sRNA, small RNA fractions were prepared from the mixed total RNA sample used for detection of PSTVd genomic RNA by Northern-blot hybridization (S3 Fig). The accumulation of PSTVd-sRNA was higher in EC than in SlRDR6i plants especially in the virulent PSTVd-RG1 isolate (Fig 6). The accumulation of PSTVd-sRNA in tomato plants was positively correlated with that of PSTVd genomic RNA. It is therefore suggested that the decrease of PSTVd accumulation is not caused by efficient RNA cleavage due to an excessive accumulation of PSTVd-sRNA.

### SlRDR6 suppression did not change the transmission rate of PSTVd through seeds in ‘Moneymaker’ tomatoes

In tomato plants, PSTVd is transmitted through seeds at a certain rate [57–60]. RDR6 suppression in *N*. *benthamiana* allows PSTVd to invade the floral meristem [33], which differentiates into various floral organs, suggesting that SlRDR6 suppression affects PSTVd transmission through seeds.

Therefore, seeds from EC and SlRDR6i plants infected with the less virulent PSTVd-Int were sowed, and the seedlings were used for the analysis of PSTVd infection by Northern-blot hybridization. The analysis revealed that PSTVd-Int was transmitted to progeny seedlings through seeds at a rate of about 1.58% (3/190) in EC plants, whereas no transmission was observed in SlRDR6i plants (0/180) (Table 1). Interestingly, the seed transmission of PSTVd-Int in EC plants was only observed in seeds from a single fruit. These results suggest that SlRDR6 suppression does not significantly increase the transmission rate of PSTVd through seeds in ‘Moneymaker’ tomatoes.

**Table 1:** SlRDR6-suppression does not change the transmission rate of PSTVd through seeds in ‘Moneymaker’ tomatoes.

Seed transmission of PSTVd-Int in transgenic tomato lines					
EC			SlRDR6i		
Fruit number	Germination rate	Transmission rate	Fruit number	Germination rate	Transmission rate
1	19/20	3/19	1	18/20	0/18
2	18/20	0/18	2	20/20	0/20
3	18/21	0/18	3	15/20	0/15
4	19/20	0/19	4	19/21	0/19
5	20/20	0/20	5	20/20	0/20
6	20/20	0/20	6	16/20	0/16
7	21/21	0/21	7	19/20	0/19
8	16/20	0/16	8	17/20	0/17
9	19/20	0/19	9	16/20	0/16
10	20/20	0/20	10	20/20	0/20
	190/202 (94.1%)	3/190 (1.58%)*		180/201 (89.6%)	0/180 (0.00%)*

*No relationship between SlRDR6-suppression and seed transmission rates of PSTVd was confirmed by Pearson's chi-square test.

## Discussion

RDR6, one of the key factors in RNA silencing, generally maintains and amplifies RNA silencing by mediating generation of dsRNA, which is the precursor of secondary siRNA. Involvement of RDR6 in defense mechanisms against virus and viroid infection has been investigated using the model plants *A*. *thaliana* and *N*. *benthamiana* [18, 23–25, 27, 32–34]. Particularly during viroid infection, RDR6 was involved in the defense mechanism based on RNA silencing against PSTVd infection in *N*. *benthamiana* [33, 34]. In the present study, an SlRDR6-suppressed ‘Moneymaker’ tomato was generated to elucidate the role of SlRDR6 in the defense response against viroid infection in tomatoes, a commercially important crop that is most sensitive to several pospiviroids, including PSTVd. SlRDR6i ‘Moneymaker’ tomato is a transgenic plant transformed with an IR-SlartRDR6 transgene composed of a set of a partial sequences of the *SlRDR6* gene placed in a head-to-head direction across an intron sequence. The expression level of endogenous *SlRDR6* is significantly reduced compared with that in EC plants (Fig 1C).

It was previously predicted that host plants deficient in PTGS may facilitate viroid replication with showing no symptoms, if RNA silencing is associated with symptom development [61]. It is reported that suppression of DCLs intensifies the severity of PSTVd symptoms in tomato or *N*. *benthamiana* plants [48, 62]. In the present study, the association of RDR6 with symptoms by PSTVd infection was investigated in tomatoes. Challenge-inoculation assay of EC and SlRDR6i plants with intermediate (isolate PSTVd-Int) and lethal (isolate PSTVd-RG1) strains of PSTVd revealed that SlRDR6-suppression in these tomato plants did not change the severity of PSTVd symptoms (Fig 2). This is consistent with a case reported previously in PSTVd-infected NbRDR6i plants [33], but not with a case of HSVd-infected plants where RDR6 was suggested to be important for symptom expression [32]. Therefore, contribution of RDR6 in the expression of disease symptoms and pathogenicity may be different depending on host-viroid combinations, and the contribution of RDR6 to the development or suppression of disease symptoms was clearly less important than that of DCLs at least in the case of PSTVd infection.

As one of the many plant defense responses against virus and viroid infection, RDR6 suppresses invasion of these pathogens to the meristem [25, 33]. In the present study, the suppression of SlRDR6 allowed two PSTVd strains to invade the SAM of ‘Moneymaker’ tomatoes at a low rate; however, the invasion was restricted to the basal part of SAM, and does not occur into the apical part of SAM including pluripotent stem cells regardless of PSTVd strain (Fig 3). The result is inconsistent in the strict sense with the previous report in NbRDR6i plants allowing the invasion of PSTVd into the whole of SAM, although the invasion rate of PSTVd into SAM of NbRDR6i plants is unknown [33]. Considering RDR6 suppression efficiency is approximately 60% in SlRDR6i plants, the suppression may not be sufficient to fully destroy the ability of RDR6 to prevent the SAM from PSTVd invasion. Alternatively, RDR6 may not be the only factor to protect SAM from PSTVd infection in tomatoes unlike *N*. *benthamiana* plants. In the case of viral infection, MtRDR1 was also reported to suppress virus entry into the SAM of *N*. *benthamiana* whose NbRDR1 was naturally defective in the function [21, 22]. In addition, the induction of RDR1 expression has been observed in ‘Rutgers’ tomatoes by PSTVd infection and ‘Suyo’ cucumbers by HSVd infection [30, 31]. Therefore, SlRDR1 may also contribute to protect SAM from PSTVd invasion in tomatoes. The report presented herein is a first study on the role of RDR6 for viroid invasion into SAM in plants possessing both functional RDR1 and RDR6.

Entry of viruses or viroids into the SAM is often associated with the development and intensification of disease symptoms [25, 33, 45, 47]. In the current study, PSTVd invasion into the SAM in SlRDR6i ‘Moneymaker’ tomato did not enhance disease symptoms and did not correlate with the virulence of PSTVd strains (Figs 2 and 3). Therefore, taking the previous reports also in consideration [33, 45, 47], these data suggest that invasion of PSTVd into the SAM is not necessarily associated with development of disease symptoms, and the difference in the pathogenicity of PSTVd strains to the tomato does not necessarily affect the extent or rate of invasion into the SAM.

The role of RDR6 in suppressing PSTVd invasion into the SAM and floral meristem [33] suggests that RDR6 may be regulating seed transmission of PSTVd. However, SlRDR6 suppression allowed PSTVd to invade the SAM to some extent while did not significantly increase the transmission rate of PSTVd through seeds in ‘Moneymaker’ tomatoes (Fig 3; Table 1). After invasion into the SAM, PSTVd may be excluded from the floral meristem during development into various floral organs as suggested in the ‘Rutgers’ tomato infected with tomato chlorotic dwarf viroid [63]. In this study, the rate of meristem invasion of PSTVd in SlRDR6i plants was low, and PSTVd did not invade the apical part of SAM regardless of PSTVd strain (Fig 3). The apical part of SAM includes the stem cells which are undifferentiated cells and serve as the origin of plant vitality, as they maintain themselves while providing a steady supply of precursor cells to form differentiated tissues and organs in plants [35–40]. Therefore, the non-invasion of PSTVd into the stem cells of SAM may result in the low seed transmission rate of PSTVd in SlRDR6i tomato plants. Furthermore, in this experiment, because the rate of fruit set in SlRDR6i ‘Moneymaker’ tomatoes became poor during PSTVd infection, the apparent seed transmission rate might have decreased. Further analysis will be needed to test whether these are the case. Moreover, we cannot exclude again the possibility that the combination of virus/viroid strain and host plant species may affect seed transmission rates [64].

It is not clear how RDR6 suppresses the invasion of virus and viroid pathogens into the SAM. Meanwhile, viral suppression of RNA silencing has allowed some viruses to enter the SAM, and CMV was excluded from SAM in *N*. *benthamiana* along with the accumulation of CMV-derived small RNA at shoot apices [41, 42]. Therefore, the pathway involving the secondary sRNA synthesis facilitated by RDR6 may not only amplify and maintain the action of RNA silencing but also may be involved in the exclusion of virus and viroid from the meristem. In SlRDR6i ‘Moneymaker’ tomato, accumulation of PSTVd genomic RNA was suppressed in early infection stage, while PSTVd invaded the SAM in the plants (Figs 3 and 4). In a previous report, it was suggested that secondary sRNA generated via dsRNA synthesis by endogenous factors such as RDRs may be functionally different from primary sRNA [26]. Because RDR6 suppression in SlRDR6i plants is also expected to reduce the amount of secondary sRNAs, reduction of secondary PSTVd-sRNA may be a cause that allows PSTVd to enter the SAM. At present, however, it is not possible to distinguish secondary sRNA from primary sRNA, so this point remains unknown.

Callose deposition on plasmodesmata (PD) raises a physical barrier restricting cell-to-cell movement of plant viruses [65]. In the case of viroid infection, suppression of CSVd entry into the SAM in *Argyranthemum* correlates with a deposition of callose (β-1,3-glucan) at the PD of the SAM, suggesting that the physical barrier of callose deposition on PD may additionally restrict the cell-to-cell movement of viroids [46, 47]. Based on these findings, callose deposition at the PD of the SAM may also be a factor for suppressing PSTVd invasion into SAM. Meanwhile, vd-sRNA derived from the virulence-modulating region of PSTVd-Int was reported to target CalS11-like and CalS12-like *callose synthase* genes of tomatoes and may suppress callose accumulation in PSTVd-infected ‘Rutgers’ tomatoes [15], while PSTVd-Int did not invade the SAM of ‘Rutgers’ tomatoes [43]. Further research is needed to determine if callose deposition at the PD in the SAM is involved in suppressing PSTVd invasion into the SAM.

RDR6 is a key factor in the secondary siRNA synthesis pathway that enhances RNA silencing. Interestingly, however, accumulation of PSTVd genomic RNA during early infection decreased in SlRDR6i ‘Moneymaker’ tomatoes, especially in PSTVd-RG1 in three independent experiments (Fig 4). It is unclear why suppression of SlRDR6 suppressed PSTVd accumulation in tomato plants. Previous reports suggest that suppression of DCL4 in *N*. *benthamiana* induces a more devastating pathway involving DCL2 and DCL3, and more strongly suppresses PSTVd accumulation [62, 66]. Given these findings, SlRDR6 suppression in tomatoes may also activate unknown hierarchical pathways and suppress PSTVd accumulation. Recently, pathogen-associated molecular patterns (PAMPs)-triggered immunity (PTI) that contributes to plant basal resistance has been discovered to act against viruses in plants, and dsRNA was shown to induce typical PTI responses by recognition as a genuine PAMP [67, 68]. Antiviral PTI induced by dsRNA is to be a distinct plant defense pathway from RNA silencing because PTI responses are not impaired in *A*. *thaliana dcl2 dcl4*, *dcl2 dcl3 dcl4* or *dcl1* mutants, although the two pathways may be functionally linked [68, 69]. Earliest signaling events in PTI responses include, for instance, the influx of Ca^2+^, the production of reactive oxygen species, and the activation of mitogen-activated protein kinase (MAPK) cascades [70]. PTI responses induced by PSTVd were suggested from comprehensive transcriptome analyses in ‘Heinz 1706’ tomatoes, and the induction of MAPK3 was verified at both transcriptional and translational levels [71]. Meanwhile, *A*. *thaliana rdr6* loss-of-function mutant was reported to exhibit constitutively activated PTI against a virulent *Pseudomonas syringae* strain; therefore, RDR6 appears to be a negative regulator of PTI [72]. Interestingly, the expression level of *SlRDR6* mRNA was significantly decreased by PSTVd infection in EC plants as if SlRDR6 was down regulated in order to activate PTI (S5 Fig). Similar phenomenon has been reported in the infection of RDV whose genome was dsRNA. RDV infection decreased the *OsRDR6* mRNA expression level in rice plants non-transformed and transformed with an empty vector or *OsRDR6* for over-expression [29]. However, the infection of RSV had no effect on the expression of *OsRDR6* in mRNA and protein levels [29]; thus, the rod-like structure of PSTVd, which resembles dsRNA to a certain extent, may affect the expression of *SlRDR6* mRNA in infection. These findings suggest that the lower accumulation of PSTVd in SlRDR6i than in EC plants during early infection may be due to PTI enhanced by the suppression of SlRDR6.

In addition to PTI, SlRDR1 also probably contributes the suppression of PSTVd accumulation because NbRDR6 suppression in *N*. *benthamiana* possessing the natural loss-of-function variant of NbRDR1 led to elevation of PSTVd accumulation even though PTI should be enhanced in *N*. *benthamiana* [33]. The NbRDR6-suppressed *N*. *benthamiana* plant is also hypersusceptible to PVX, PVY, and CMV in combination with the Y satellite but not to TMV, TRV, TCV, or CMV alone [25, 27]. Efficient RNA silencing driven by PVX and PPV VIGS-vectors is dependent upon NbRDR6 but that driven by TRV-vector is not in NbRDR6-suppressed *N*. *benthamiana* plants [26]. Likewise, *A*. *thaliana rdr6* mutant is hypersusceptible to CMV but not to TMV, TRV, turnip mosaic virus, or turnip vein clearing virus [18, 73, 23]. Considering these findings, the accumulation of TMV and TRV is independent upon the suppression of RDR6 in both *N*. *benthamiana* and *A*. *thaliana*, whereas the accumulation of CMV is depend on the suppression of RDR6 in *A*. *thaliana* but not in *N*. *benthamiana*. Namely, the influence of RDR6 suppression on the accumulation of virus is dependent upon virus species and/or the host plant, and host factors such as other RDRs rather than RDR6 may contribute effective RNA silencing for several viruses such as TMV and TRV. In *A*. *thaliana*, TRV-derived siRNA biogenesis and antiviral silencing are strongly dependent upon the combined activity of RDR1, RDR2, and RDR6 [74]. Therefore, SlRDR(s) other than SlRDR6, such as SlRDR1, probably contributes to the production of virus-derived secondary siRNAs [75] which could induce more effective RNA silencing than primary siRNAs [26]. In the present study, the expression level of *SlRDR1* mRNA in the infection with PSTVd, especially with PSTVd-RG1, in SlRDR6i plants was higher than that in EC plants at 15 dpi, suggesting that the suppression of SlRDR6 resulted in the activation of *SlRDR1* expression, and correlating negatively with the accumulation of PSTVd (Figs 4 and 5; S7 Fig). The suppression of PSTVd accumulation in SlRDR6i tomato plants during early infection appears at first glance to contradict the findings of a previous report on PSTVd-infected NbRDR6i plants [33], but the potential effect of RDR6 suppression on viroid accumulation depending on each host-viroid combination as well as deficiency of RDR1 in *N*. *benthamiana* may contribute to this discrepancy [21]. It has been suggested that RDR1 of *N*. *benthamiana* may have become defective due to strong selective pressure by the RDR6-mediated anti-virus system that acquired full activity through co-evolution during the long-term virus-host arms race [76]. In the future, it will be necessary to examine the role of RDR1 upon viroid infection.

In conclusion, RNAi-mediated suppression of SlRDR6 in the ‘Moneymaker’ tomato, a cultivar having a tolerance for PSTVd infection, did not alter this tolerance against PSTVd infection. It did, however, increase the degree and rate of PSTVd invasion into SAM regardless of PSTVd strain. The invasion was restricted to the basal part of SAM and did not occur the apical part including pluripotent stem cells unlike the case of NbRDR6i plants. The invasion of PSTVd into the SAM in SlRDR6-suppressed tomatoes may not depend on PSTVd virulence or concentration because the invasion occurred similar degree and rate in two different pathogenic strains and the accumulation of PSTVd genomic and small RNAs in SlRDR6i plants decreased rather than increased in the two strains compared to that in EC plants. In addition, SlRDR6 suppression in tomato plants did not affect the seed transmission rate of PSTVd. Considering these findings comprehensively, therefore, SlRDR6 plays an important role in suppressing PSTVd entry into the basal part of SAM in tomato plants as well as *N*. *benthamiana*; however, unlike *N*. *benthamiana*, SlRDR6 is not the only factor and other factors such as SlRDR1 may also contribute to protect SAM, especially the apical part including pluripotent stem cells, from PSTVd infection in tomatoes. The SlRDR1 also probably contributes effective RNA silencing causing the decrease of PSTVd accumulation during early infection in tomato plants.

## Supporting information

S1 Fig Diagram of the SlartRDR6 transgene introduced into line 91B. The chimeric sequence of SlartRDR6 was designed from parts of *SlRDR6a* and *SlRDR6b* mRNA sequences and was used for inverted repeat (IR) construction containing an intron sequence. The IR-SlartRDR6 construct was inserted downstream of the CaMV-35S promoter and upstream of the NOS terminator.
(PDF)

S2 Fig Target region of DIG-labeled cRNA probe for CaMV-35S promoter. (PDF)

S3 Fig How to adjust total RNA samples used for RT-qPCR and Northern-blot hybridization. (PDF)

S4 Fig Amplification of a partial sequence of transgene 35S promoter by PCR and confirmation of the copy number of transgenes by Southern-blot hybridization. (A) Detection of the 35S promoter sequence was performed using two individuals in each transgenic tomato line. The primer sets used for PCR are described in S1 Table. An amplified fragment derived from the 35S promoter sequence was detected only in line 91B. (B) The copy number of transgenes was confirmed by Southern-blot hybridization using a DIG-labeled cRNA probe for the CaMV-35S promoter. A single band in both *Bam*HI and *Eco*RI digestions was detected only in line 91B.
(PDF)

S5 Fig Time-course analysis of *SlRDR6* expression levels. The expression levels of endogenous *SlRDR6* mRNA were analyzed by RT-qPCR. qPCR analysis was performed with the PCR primers for endogenous *SlRDR6* mRNA. Mean values are based on three biological replicates of the pooled sample of five individual plants. The relative expression levels were calculated for each time point with the value of EC plants inoculated with mock as a standard. The expression level of endogenous *SlRDR6* mRNA in Mock-inoculated SlRDR6i plants decreased to approximately 50% of that in Mock-inoculated EC plants. In addition, PSTVd infection tended to decrease the *SlRDR6* expression level.
(PDF)

S6 Fig Time-course analysis of *SlRDR1* expression levels. The expression levels of endogenous *SlRDR1* mRNA were analyzed by RT-qPCR. qPCR analysis was performed with the PCR primers for endogenous *SlRDR1* mRNA. Mean values are based on three biological replicates of the pooled sample of five individual plants. The relative expression levels were calculated for each time point with the value of EC plants inoculated with mock as a standard. The expression levels of endogenous *SlRDR1* mRNA were apparently different between Int- or RG1-infected SlRDR6i plants at later infection stage, or between RG1-infected EC and SlRDR6i plants at 15 dpi.
(PDF)

S7 Fig Time-course analysis of PSTVd accumulation by Northern-blot hybridization and RT-qPCR (one of the repeated tests). (A) Accumulation of PSTVd genomic RNA was analyzed by Northern-blot hybridization with DIG-labeled cRNA probe for PSTVd. Each lane was loaded with each total RNA sample extracted from pooled five leaf disks collected from five individual plants. rRNAs were stained with ethidium bromide and used as a loading control. At 15 dpi, the accumulation of PSTVd-RG1 was lower in SlRDR6i plants than in EC plants. (B) Accumulation levels of PSTVd genomic RNA were also analyzed by RT-qPCR. qPCR analysis was performed with the PCR primers for PSTVd. Mean values are based on three biological replicates of the total RNA sample from five individual plants. The relative PSTVd levels were calculated for each time point with the value of EC plants inoculated with PSTVd-Int as a standard. At 5 and 10 dpi, at which the accumulation of PSTVd was not detectable by Northern-blot hybridization, the accumulation levels of PSTVd-Int increased in SlRDR6i plants compared to that in EC plants, while those of PSTVd-RG1 decreased in SlRDR6i plants. The statistically significant difference of PSTVd accumulation was confirmed by Welch’s or Student’s t-test. (C) The line graphs indicate time-course changes in the accumulation levels of PSTVd-Int or PSTVd-RG1. The relative PSTVd levels were calculated with the value of EC plants inoculated with PSTVd-Int at 5 dpi as a standard. During 10–15 dpi, the accumulation levels of PSTVd-Int were reversed between EC and SlRDR6i plants (The reverse point is indicated with a red arrow).
(PDF)

S1 Table The list of primers used in PCR and RT-qPCR. (PDF)

S1 Raw Images (PDF)
