# Supplementary material for: Suppression of RNA-dependent RNA polymerase 6 in tomatoes allows potato spindle tuber viroid to invade basal part but not apical part including pluripotent stem cells of shoot apical meristem
Source: PLoS One. 2020 Jul 27;15(7):e0236481. doi: 10.1371/journal.pone.0236481 (PMC7384629; doi:10.1371/journal.pone.0236481)
Supplement: S1 Fig — The chimeric sequence of SlartRDR6 was designed from parts of SlRDR6a and SlRDR6b mRNA sequences and was used for inverted repeat (IR) construction containing an intron sequence. The IR-SlartRDR6 construct was inserted downstream of the CaMV-35S promoter and upstream of the NOS terminator. (PDF) [file pone.0236481.s001.pdf]

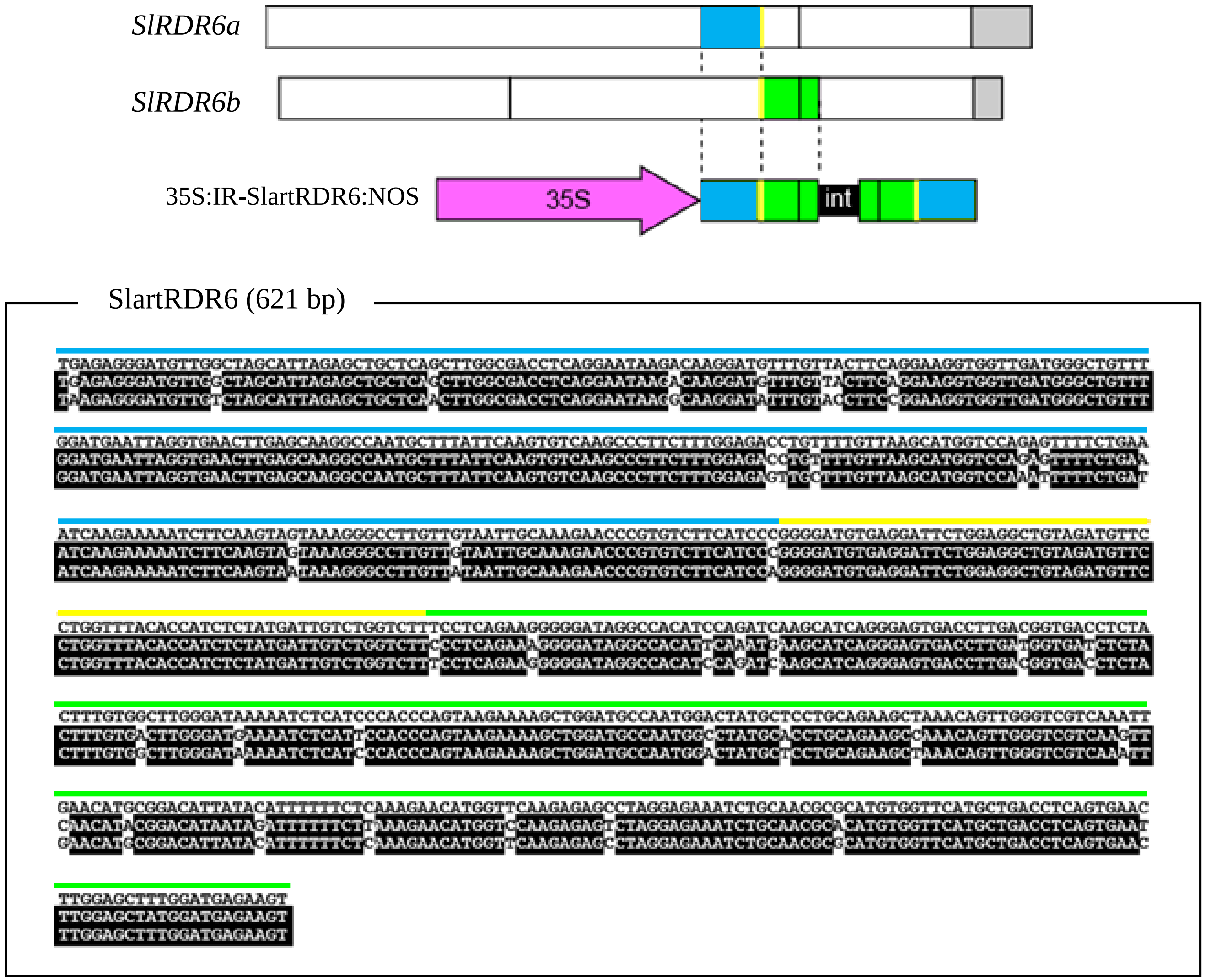
SlRDR6a
SlRDR6b
35S:IR-SlartRDR6:NOS
35S
int
SlartRDR6 (621 bp)

S1 Fig. Diagram of the SlartRDR6 transgene introduced into line 91B. The chimeric sequence of SlartRDR6 was designed from parts of *SlRDR6a* and *SlRDR6b* mRNA sequences and was used for inverted repeat (IR) construction containing an intron sequence. The IR-SlartRDR6 construct was inserted downstream of the CaMV-35S promoter and upstream of the NOS terminator.
