# Supplementary material for: Suppression of RNA-dependent RNA polymerase 6 in tomatoes allows potato spindle tuber viroid to invade basal part but not apical part including pluripotent stem cells of shoot apical meristem
Source: PLoS One. 2020 Jul 27;15(7):e0236481. doi: 10.1371/journal.pone.0236481 (PMC7384629; doi:10.1371/journal.pone.0236481)
Supplement: S2 Fig — (PDF) [file pone.0236481.s002.pdf]

CaMV-35S promoter sequence (835 bp)

AGATTAGCCTTTTCAATTTCAGAAAGAATGCTAACCCACAGATGGTTAGAGAGGCTTACGCAG
CAGGTCTCATCAAGACGATCTACCCGAGCAATAATCTCCAGGAAATCAAATACCTTCCCAAGA
AGGTTAAAGATGCAGTCAAAAGATTCAGGACTAACTGCATCAAGAACACAGAGAAAGATATA
TTTCTCAAGATCAGAAGTACTATTCCAGTATGGACGATTCAAGGCTTGCTTCACAAACCAAGG
CAAGTAATAGAGATTGGAGTCTCTAAAAAGGTAGTTCCCACTGAATCAAAGGCCATGGAGTC
AAAGATTCAAATAGAGGACCTAACAGAACTCGCCGTAAAGACTGGCGAACAGTTCATACAGA
GTCTCTTACGACTCAATGACAAGAAGAAAATCTTCGTCAACATGGTGGAGCACGACACACTT
GTCTACTCCAAAAATATCAAAGATACAGTCTCAGAAGACCAAAGGGCAATTGAGACTTTTCA
ACAAAGGGTAATATCCGGAAACCTCCTCGGATTCCATTGCCCAGCTATCTGTCACTTTATTGTG
AAGATAGTGGAAAAGGAAGGTGGCTCCTACAAATGCCATCATTGCGATAAAGGAAAGGCCAT
CGTTGAAGATGCCTCTGCCGACAGTGGTCCCAAAGATGGACCCCCACCCACGAGGAGCATCG
TGGAAAAAGAAGACGTTCCAACCACGTCTTCAAAGCAAGTGGATTGATGTGATATCTCCACT
GACGTAAGGGATGACGCACAATCCCACTATCCTTCGCAAGACCCTTCCTCTATATAAGGAAGT
TCATTTCATTTGGAGAGAACACG

Target region of DIG-labeled cRNA probe for 35S promoter is indicate with yellow background.

S2 Fig. Target region of DIG-labeled cRNA probe for CaMV-35S promoter.
