# Supplementary material for: Suppression of RNA-dependent RNA polymerase 6 in tomatoes allows potato spindle tuber viroid to invade basal part but not apical part including pluripotent stem cells of shoot apical meristem
Source: PLoS One. 2020 Jul 27;15(7):e0236481. doi: 10.1371/journal.pone.0236481 (PMC7384629; doi:10.1371/journal.pone.0236481)
Supplement: S3 Fig — (PDF) [file pone.0236481.s003.pdf]

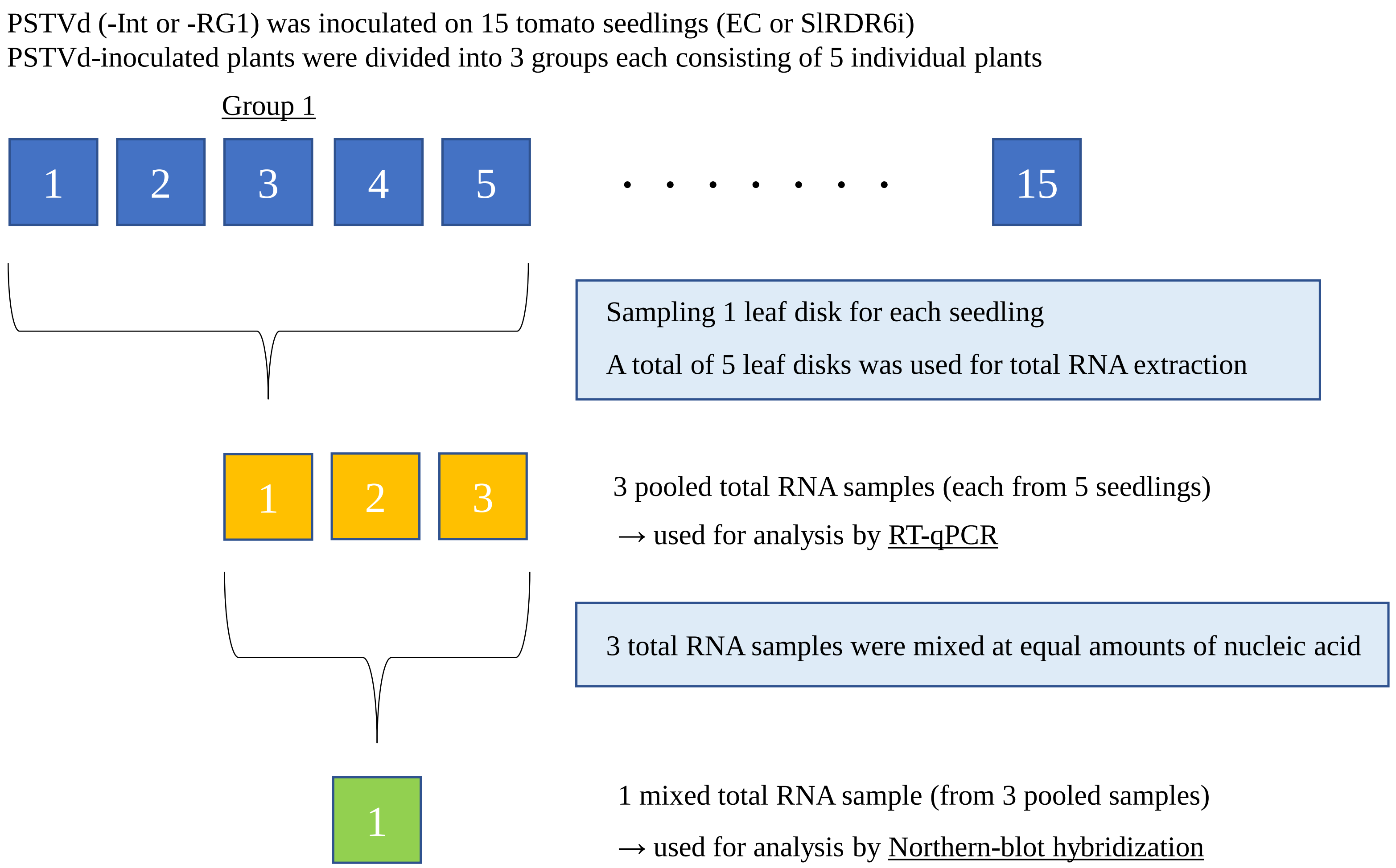

S3 Fig. How to adjust total RNA samples used for RT-qPCR and Northern-blot hybridization.
