# Supplementary material for: Suppression of RNA-dependent RNA polymerase 6 in tomatoes allows potato spindle tuber viroid to invade basal part but not apical part including pluripotent stem cells of shoot apical meristem
Source: PLoS One. 2020 Jul 27;15(7):e0236481. doi: 10.1371/journal.pone.0236481 (PMC7384629; doi:10.1371/journal.pone.0236481)
Supplement: S4 Fig — (A) Detection of the 35S promoter sequence was performed using two individuals in each transgenic tomato line. The primer sets used for PCR are described in S1 Table. An amplified fragment derived from the 35S promoter sequence was detected only in line 91B. (B) The copy number of transgenes was confirmed by Southern-blot hybridization using a DIG-labeled cRNA probe for the CaMV-35S promoter. A single band in both BamHI and EcoRI digestions was detected only in line 91B. (PDF) [file pone.0236481.s004.pdf]

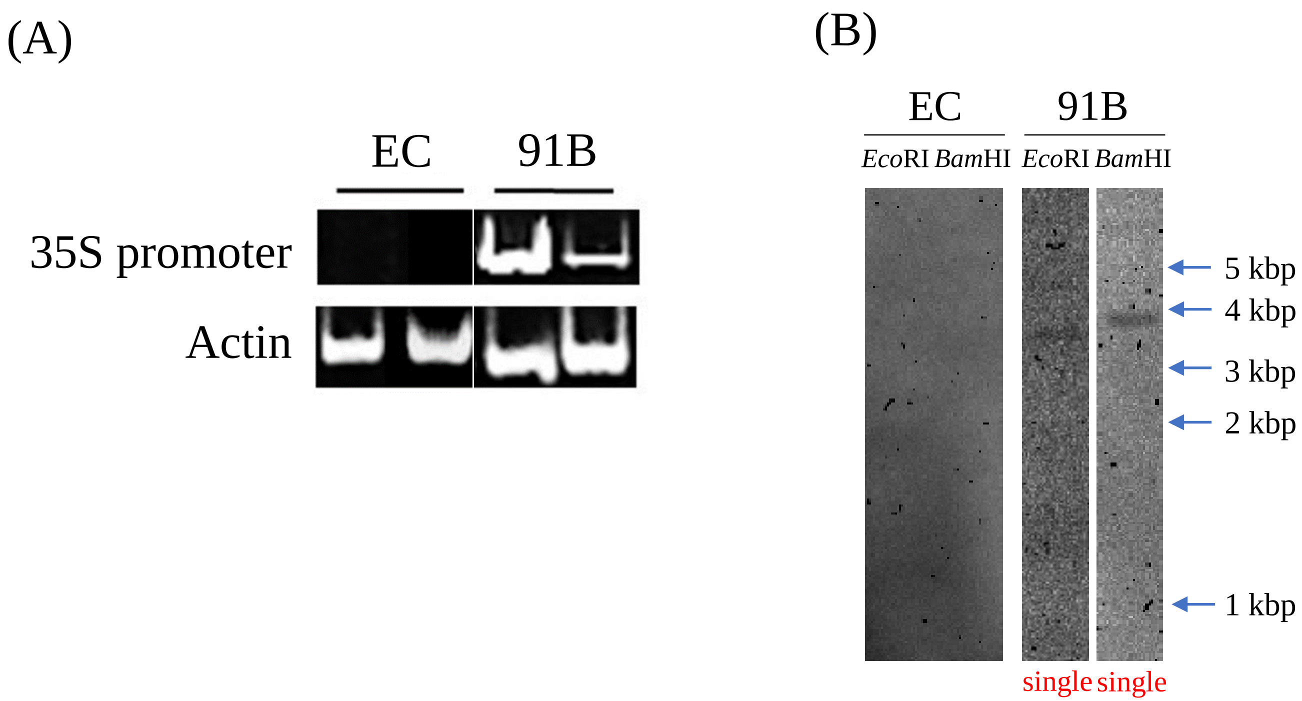
(A)
EC
91B
35S promoter
Actin
(B)
EC
91B
EcoRI BamHI EcoRI BamHI
5 kbp
4 kbp
3 kbp
2 kbp
1 kbp
single single

S4 Fig. Amplification of a partial sequence of transgene 35S promoter by PCR and confirmation of the copy number of transgenes by Southern-blot hybridization. (A) Detection of the 35S promoter sequence was performed using two individuals in each transgenic tomato line. The primer sets used for PCR are described in Table S1. An amplified fragment derived from the 35S promoter sequence was detected only in line 91B. (B) The copy number of transgene was confirmed by Southern-blot hybridization using a DIG-labeled cRNA probe for the CaMV-35S promoter. A single band in both *Bam*HI and *Eco*RI digestions was detected only in line 91B.
