# Supplementary material for: Suppression of RNA-dependent RNA polymerase 6 in tomatoes allows potato spindle tuber viroid to invade basal part but not apical part including pluripotent stem cells of shoot apical meristem
Source: PLoS One. 2020 Jul 27;15(7):e0236481. doi: 10.1371/journal.pone.0236481 (PMC7384629; doi:10.1371/journal.pone.0236481)
Supplement: S5 Fig — The expression levels of endogenous SlRDR6 mRNA were analyzed by RT-qPCR. qPCR analysis was performed with the PCR primers for endogenous SlRDR6 mRNA. Mean values are based on three biological replicates of the pooled sample of five individual plants. The relative expression levels were calculated for each time point with the value of EC plants inoculated with mock as a standard. The expression level of endogenous SlRDR6 mRNA in Mock-inoculated SlRDR6i plants decreased to approximately 50% of that in Mock-inoculated EC plants. In addition, PSTVd infection tended to decrease the SlRDR6 expression level. (PDF) [file pone.0236481.s005.pdf]

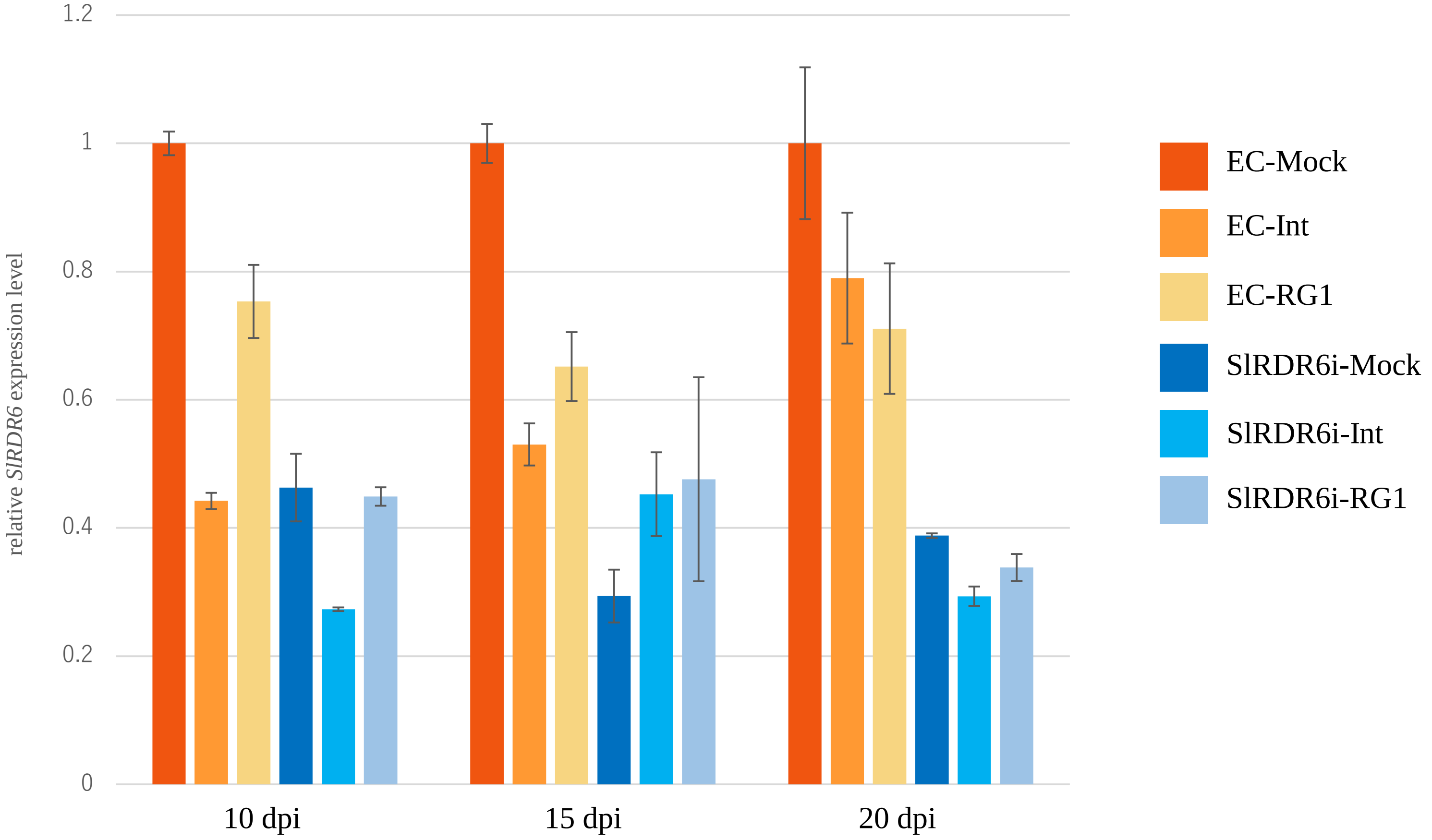

1.2
1
0.8
0.6
0.4
0.2
0
relative *SlRDR6* expression level
10 dpi
15 dpi
20 dpi
EC-Mock
EC-Int
EC-RG1
SlRDR6i-Mock
SlRDR6i-Int
SlRDR6i-RG1

S5 Fig. Time-course analysis of *SlRDR6* expression levels. The expression levels of endogenous *SlRDR6* mRNA were analyzed by RT-qPCR. qPCR analysis was performed with the PCR primers for endogenous *SlRDR6* mRNA. Mean values are based on three biological replicates of the pooled sample of five individual plants. The relative expression levels were calculated for each time point with the value of EC plants inoculated with mock as a standard. The expression level of endogenous *SlRDR6* mRNA in Mock-inoculated SlRDR6i plants decreased to approximately 50% of that in Mock-inoculated EC plants. In addition, PSTVd infection tended to decrease the *SlRDR6* expression level.
