# Supplementary material for: Suppression of RNA-dependent RNA polymerase 6 in tomatoes allows potato spindle tuber viroid to invade basal part but not apical part including pluripotent stem cells of shoot apical meristem
Source: PLoS One. 2020 Jul 27;15(7):e0236481. doi: 10.1371/journal.pone.0236481 (PMC7384629; doi:10.1371/journal.pone.0236481)
Supplement: S6 Fig — The expression levels of endogenous SlRDR1 mRNA were analyzed by RT-qPCR. qPCR analysis was performed with the PCR primers for endogenous SlRDR1 mRNA. Mean values are based on three biological replicates of the pooled sample of five individual plants. The relative expression levels were calculated for each time point with the value of EC plants inoculated with mock as a standard. The expression levels of endogenous SlRDR1 mRNA were apparently different between Int- or RG1-infected SlRDR6i plants at later infection stage, or between RG1-infected EC and SlRDR6i plants at 15 dpi. (PDF) [file pone.0236481.s006.pdf]

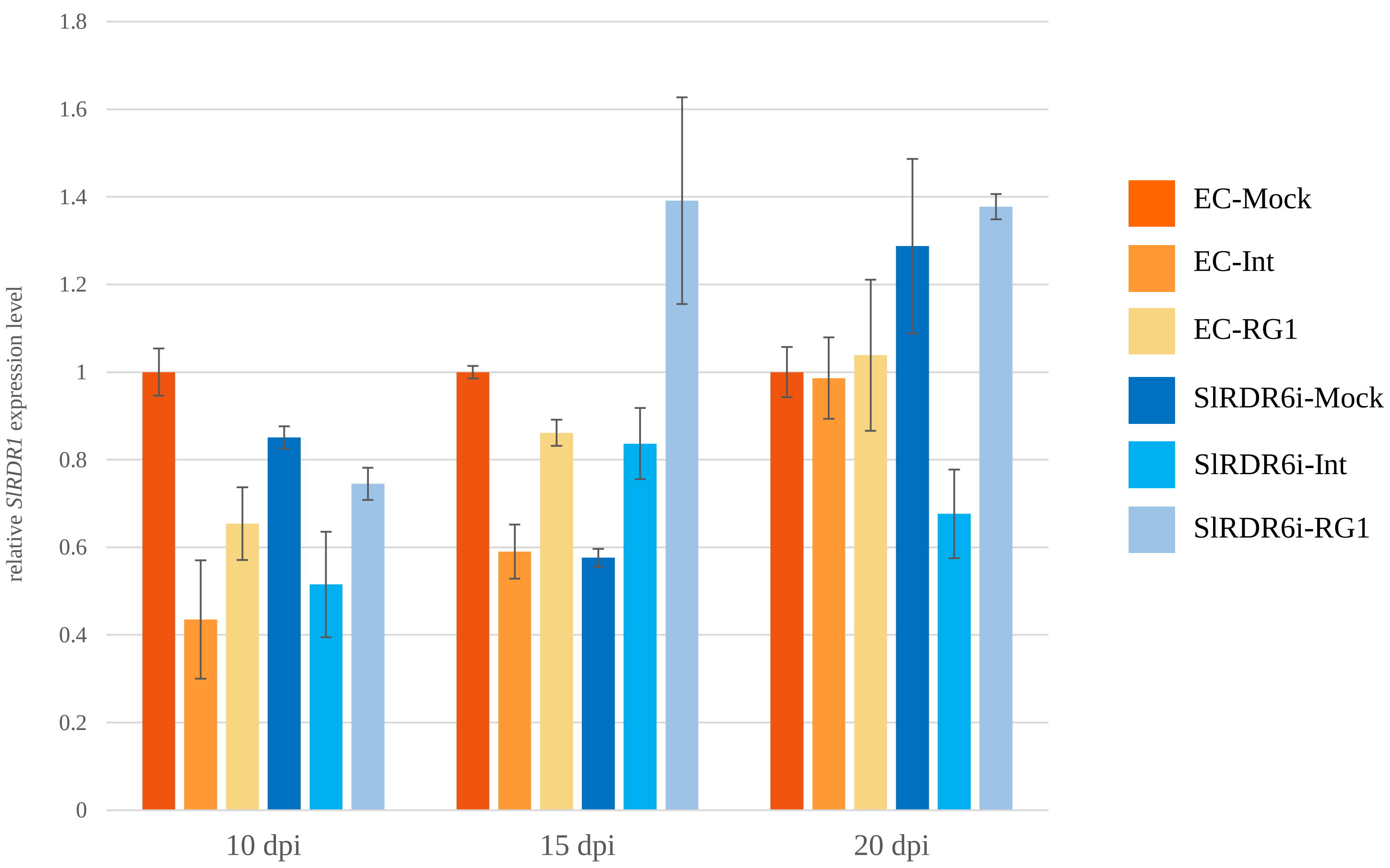
relative *SlRDR1* expression level
1.8
1.6
1.4
1.2
1
0.8
0.6
0.4
0.2
0
10 dpi
15 dpi
20 dpi
EC-Mock
EC-Int
EC-RG1
SlRDR6i-Mock
SlRDR6i-Int
SlRDR6i-RG1

S6 Fig. Time-course analysis of *SlRDR1* expression levels. The expression levels of endogenous *SlRDR1* mRNA were analyzed by RT-qPCR. qPCR analysis was performed with the PCR primers for endogenous *SlRDR1* mRNA. Mean values are based on three biological replicates of the pooled sample of five individual plants. The relative expression levels were calculated for each time point with the value of EC plants inoculated with mock as a standard. The expression levels of endogenous *SlRDR1* mRNA were apparently different between Int- or RG1-infected SlRDR6i plants at later infection stage, or between RG1-infected EC and SlRDR6i plants at 15 dpi.
