# Supplementary material for: Suppression of RNA-dependent RNA polymerase 6 in tomatoes allows potato spindle tuber viroid to invade basal part but not apical part including pluripotent stem cells of shoot apical meristem
Source: PLoS One. 2020 Jul 27;15(7):e0236481. doi: 10.1371/journal.pone.0236481 (PMC7384629; doi:10.1371/journal.pone.0236481)
Supplement: S7 Fig — (A) Accumulation of PSTVd genomic RNA was analyzed by Northern-blot hybridization with DIG-labeled cRNA probe for PSTVd. Each lane was loaded with each total RNA sample extracted from pooled five leaf disks collected from five individual plants. rRNAs were stained with ethidium bromide and used as a loading control. At 15 dpi, the accumulation of PSTVd-RG1 was lower in SlRDR6i plants than in EC plants. (B) Accumulation levels of PSTVd genomic RNA were also analyzed by RT-qPCR. qPCR analysis was performed with the PCR primers for PSTVd. Mean values are based on three biological replicates of the total RNA sample from five individual plants. The relative PSTVd levels were calculated for each time point with the value of EC plants inoculated with PSTVd-Int as a standard. At 5 and 10 dpi, at which the accumulation of PSTVd was not detectable by Northern-blot hybridization, the accumulation levels of PSTVd-Int increased in SlRDR6i plants compared to that in EC plants, while those of PSTVd-RG1 decreased in SlRDR6i plants. The statistically significant difference of PSTVd accumulation was confirmed by Welch’s or Student’s t-test. (C) The line graphs indicate time-course changes in the accumulation levels of PSTVd-Int or PSTVd-RG1. The relative PSTVd levels were calculated with the value of EC plants inoculated with PSTVd-Int at 5 dpi as a standard. During 10–15 dpi, the accumulation levels of PSTVd-Int were reversed between EC and SlRDR6i plants (The reverse point is indicated with a red arrow). (PDF) [file pone.0236481.s007.pdf]

(A)

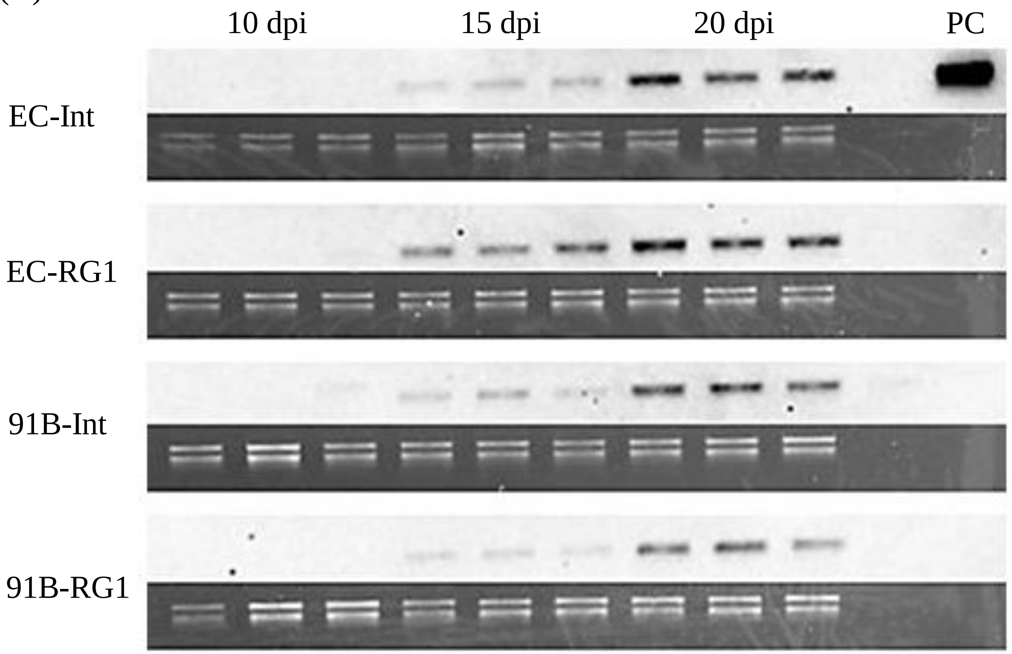

PC; positive control

(B)

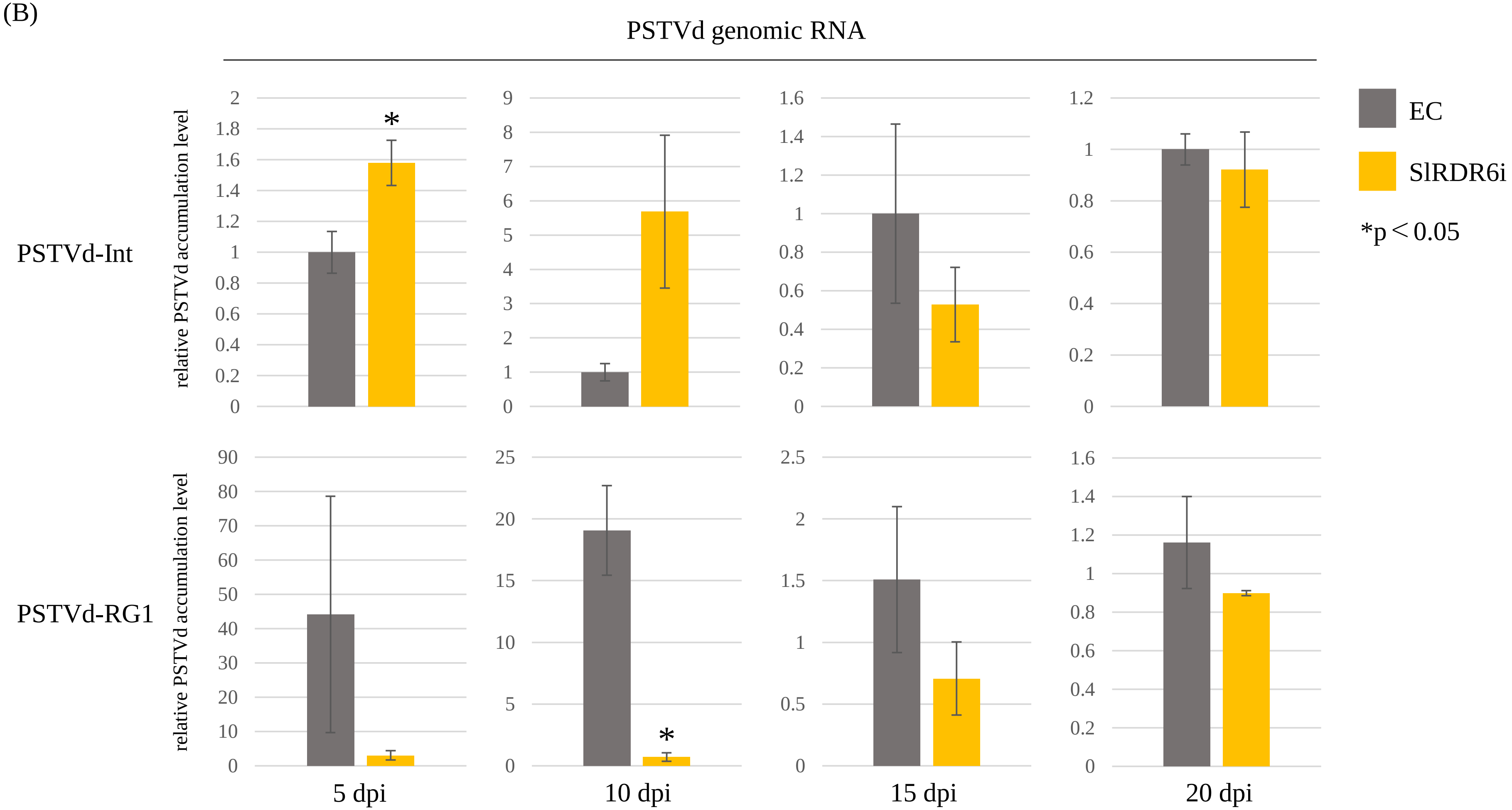
PSTVd genomic RNA
PSTVd-Int
PSTVd-RG1
relative PSTVd accumulation level
5 dpi
10 dpi
15 dpi
20 dpi
EC
SlRDR6i
*p < 0.05

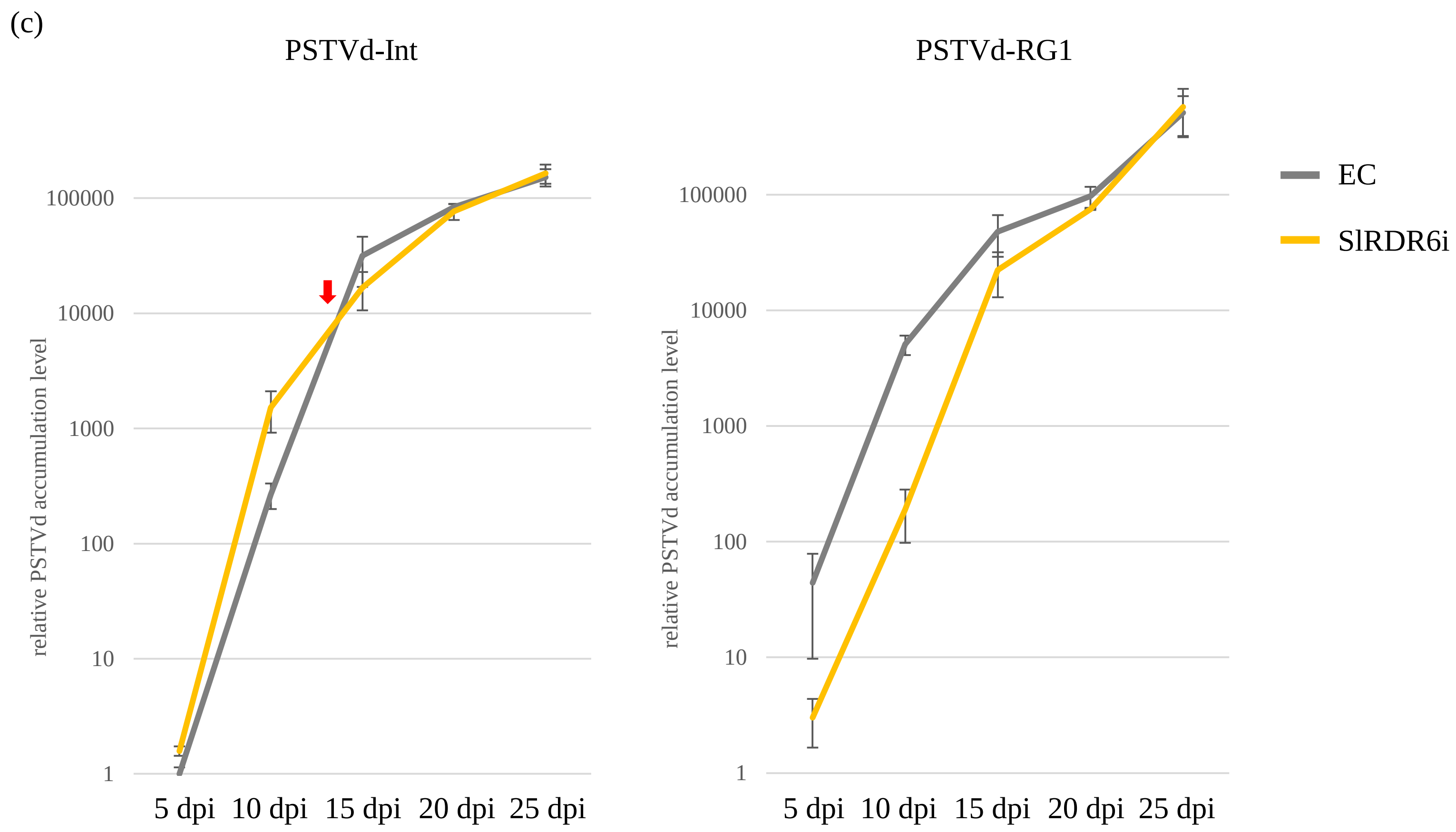

(c)
PSTVd-Int
PSTVd-RG1
relative PSTVd accumulation level
100000
10000
1000
100
10
1
5 dpi
10 dpi
15 dpi
20 dpi
25 dpi
EC
SlRDR6i

S7 Fig. Time-course analysis of PSTVd accumulation by Northern-blot hybridization and RT-qPCR (one of the repeated tests). (A) Accumulation of PSTVd genomic RNA was analyzed by Northern-blot hybridization with DIG-labeled cRNA probe for PSTVd. Each lane was loaded with each total RNA sample extracted from pooled five leaf dicks collected from five individual plants. rRNAs were stained with ethidium bromide and used as a loading control. At 15 dpi, the accumulation of PSTVd-RG1 was lower in SlRDR6i plants than in EC plants. (B) Accumulation levels of PSTVd genomic RNA were also analyzed by RT-qPCR. qPCR analysis was performed with the PCR primers for PSTVd. Mean values are based on three biological replicates of the total RNA sample from five individual plants. The relative PSTVd levels were calculated for each time point with the value of EC plants inoculated with PSTVd-Int as a standard. At 5 and 10 dpi, at which the accumulation of PSTVd was not detectable by Northern-blot hybridization, the accumulation levels of PSTVd-Int increased in SlRDR6i plants compared to that in EC plants, while those of PSTVd-RG1 decreased in SlRDR6i plants. The statistically significant difference of PSTVd accumulation was confirmed by Welch’s or Student’s t-test. (C) The line graphs indicate time-course changes in the accumulation levels of PSTVd-Int or PSTVd-RG1. The relative PSTVd levels were calculated with the value of EC plants inoculated with PSTVd-Int at 5 dpi as a standard. During 10-15 dpi, the accumulation levels of PSTVd-Int were reversed between EC and SlRDR6i plants (The reverse point is indicated with a red arrow).
