# Supplementary material for: Suppression of RNA-dependent RNA polymerase 6 in tomatoes allows potato spindle tuber viroid to invade basal part but not apical part including pluripotent stem cells of shoot apical meristem
Source: PLoS One. 2020 Jul 27;15(7):e0236481. doi: 10.1371/journal.pone.0236481 (PMC7384629; doi:10.1371/journal.pone.0236481)
Supplement: S1 Table — (PDF) [file pone.0236481.s008.pdf]

S1 Table. The list of primers used in PCR and RT-qPCR.

Target	Primer name	Primer sequence 5'–3'	Reference
SlRDR1	RDR1-For	ATGCTGAGGCCATTAGTGTTGCTG	[41]
	RDR1-Rev	CCAAGCCGAAGCCTTTGGTAACAT	
SlRDR6	RDR6-For	GCGGCTATAATGTTGAGTGCAGGG	[41]
	RDR6-Rev	GTCTTATTCCTGAGGTCGCCAAGC	
PSTVd	PSTVd-231F	GCCCCCTTTGCGCTGT	[14]
	PSTVd-296R	AAGCGGTTCTCGGGAGCTT	
β-actin	β-actin F	GAGGACAGGATGCTCCTCAG	[This study]
	β-actin R	AGACGCCTATGTGGGAGATG	
35S promoter	35S-For	AGGAAACAGCTATGACCATG	[37]
	35S-Rev	GAACTTCCTTATATAGAGGAAGG	
actin	actin-For	GCCATGCCCTTTTAAATGGC	[37]
	actin-Rev	CAGTTGGAGGGGACTGAATC	
