# Supplementary material for: Suppression of RNA-dependent RNA polymerase 6 in tomatoes allows potato spindle tuber viroid to invade basal part but not apical part including pluripotent stem cells of shoot apical meristem
Source: PLoS One. 2020 Jul 27;15(7):e0236481. doi: 10.1371/journal.pone.0236481 (PMC7384629; doi:10.1371/journal.pone.0236481)
Supplement: S1 Raw Images — (PDF) [file pone.0236481.s009.pdf]

Fig. 1A gel and blot images

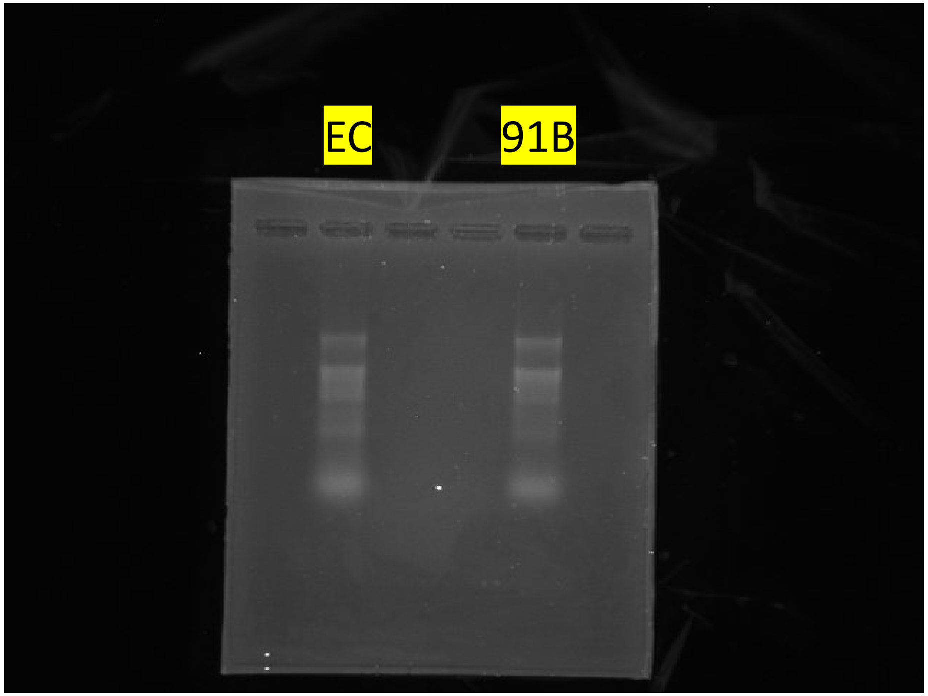

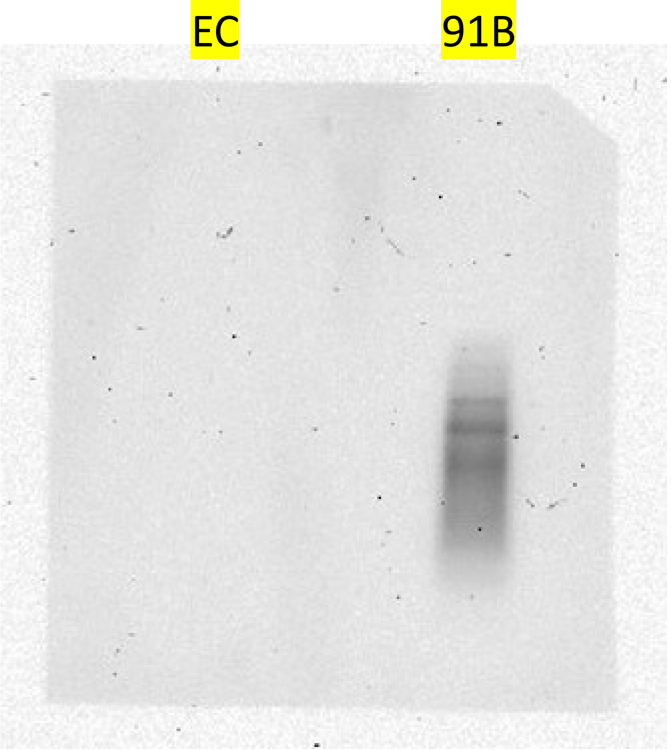

Detection of transcripts derived from transgene by Northern hybridization

Fig. 1B gel and blot images

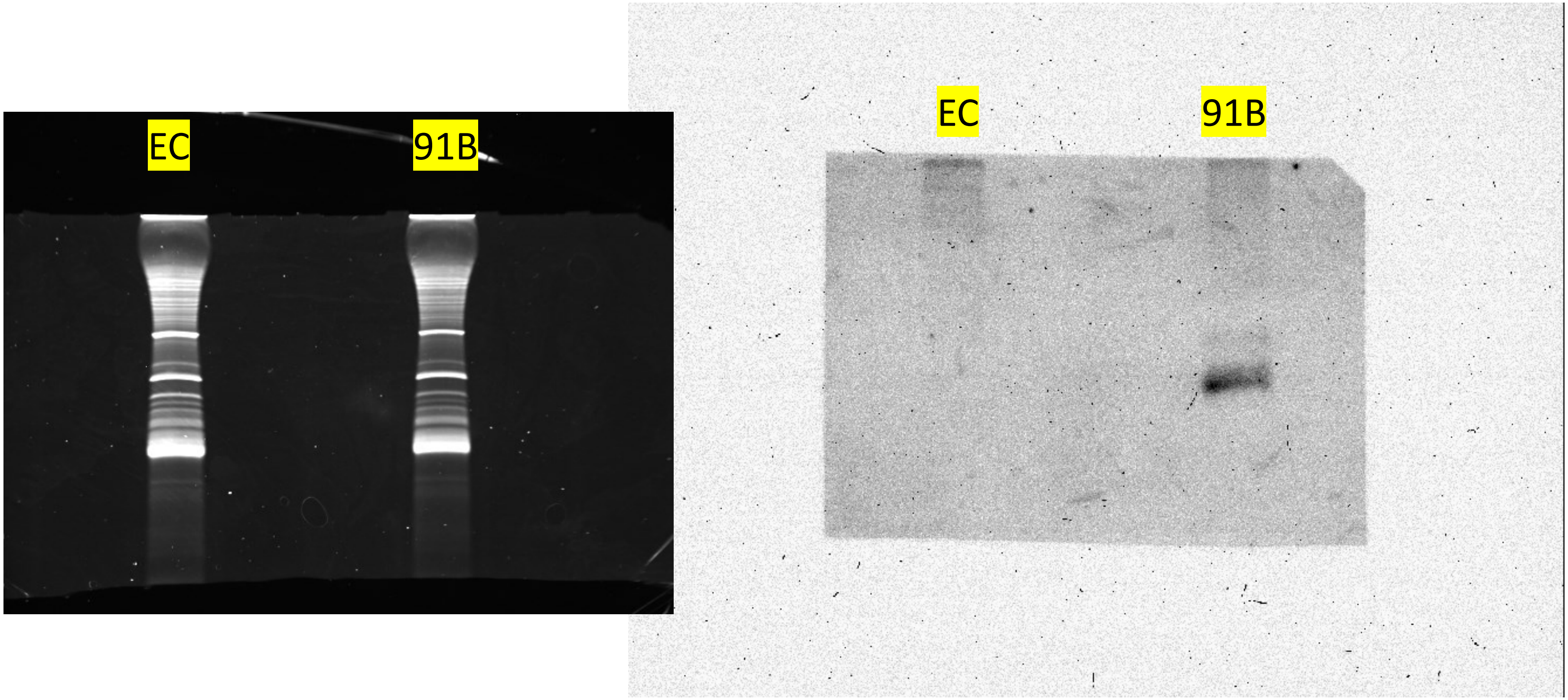

Detection of transgene-derived small RNA by Northern hybridization

Fig. 4A gel and blot images-1

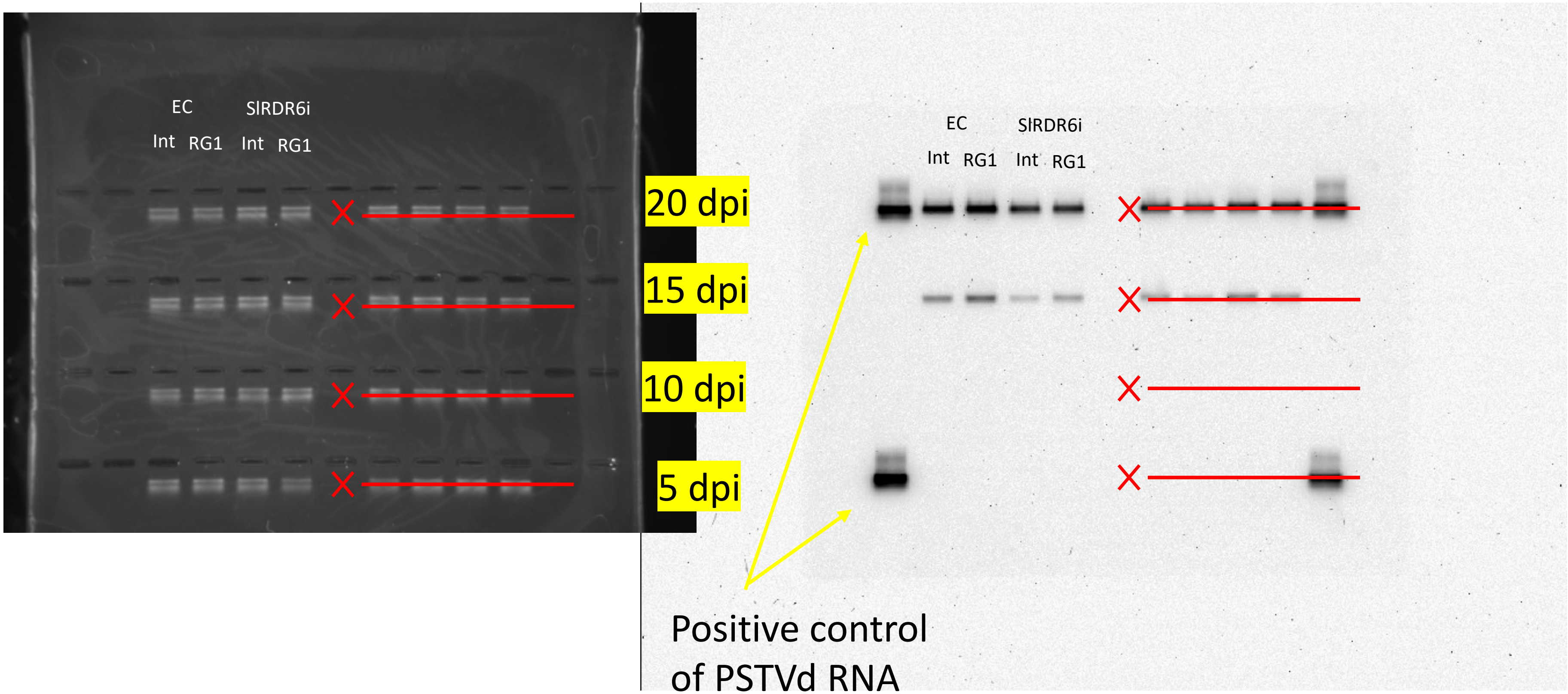

Detection of PSTVd RNA by Northern hybridization

Fig. 4A gel and blot images-2

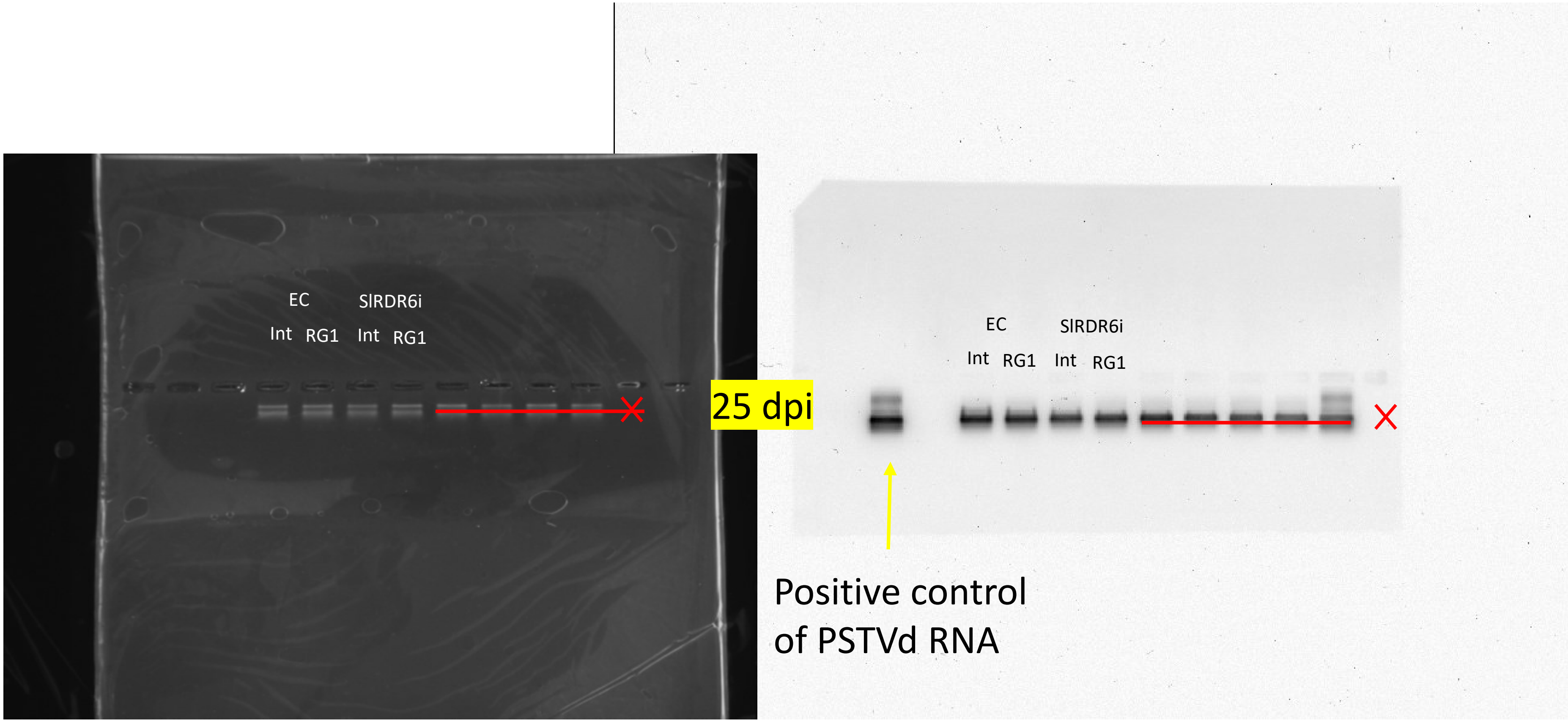

Detection of PSTVd RNA by Northern hybridization

Fig. 6 gel and blot images

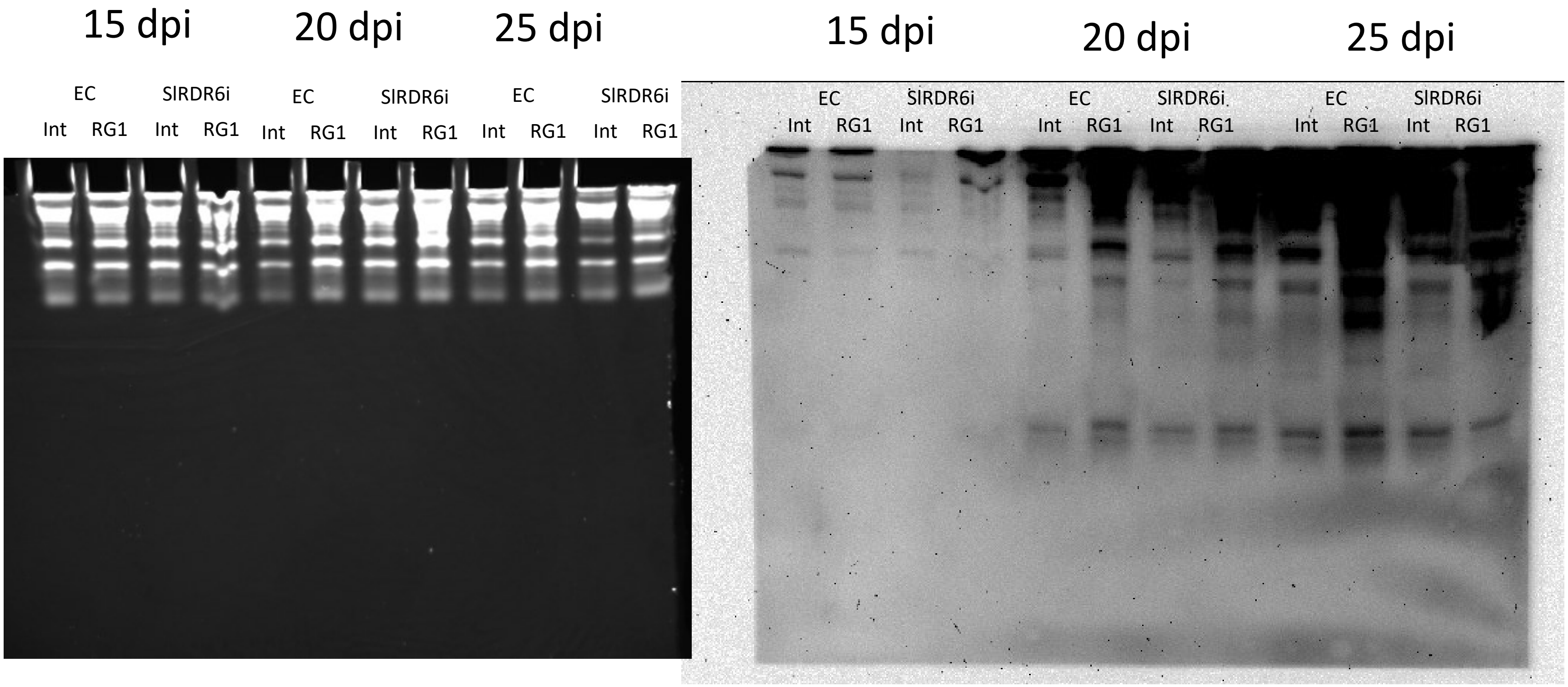

Detection of PSTVd-derived small RNA by Northern hybridization

S4A Fig. gel images

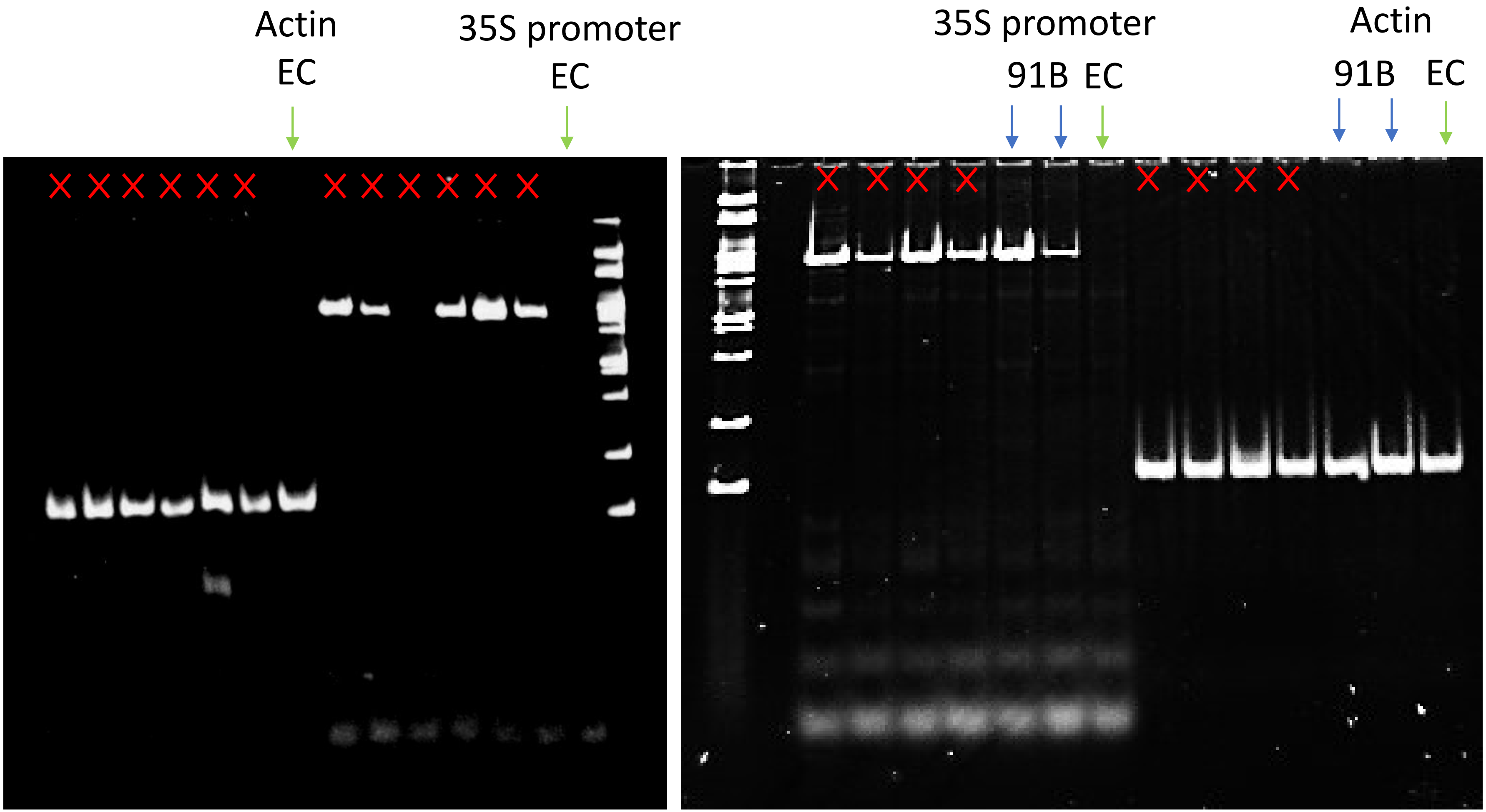

Amplification and detection of a part of transgene and actin gene by PCR and agarose gel electrophoresis.

S4B Fig. blot images-1

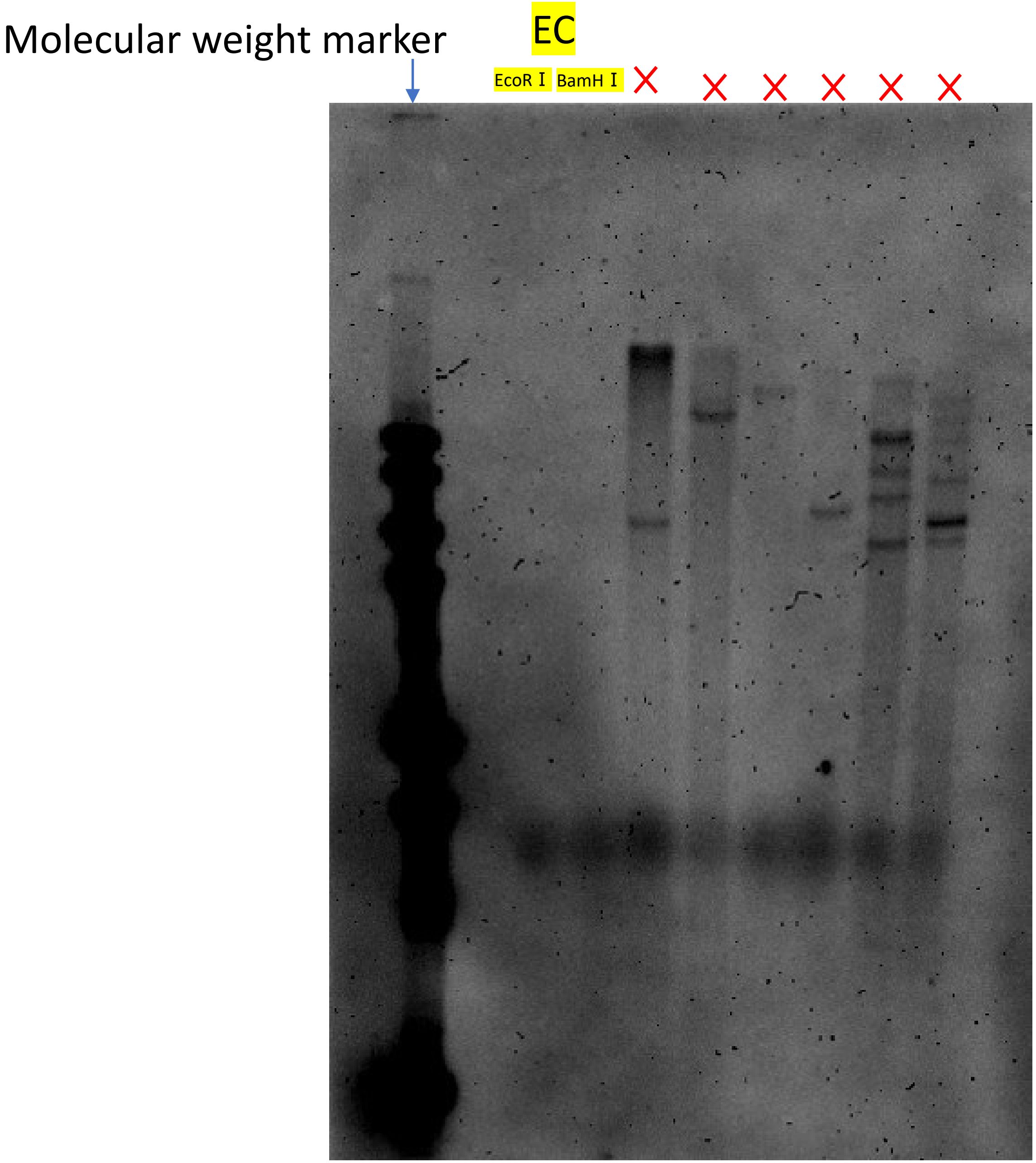

Confirmation of the copy number of transgene by Southern hybridization

S4B Fig. blot images-2

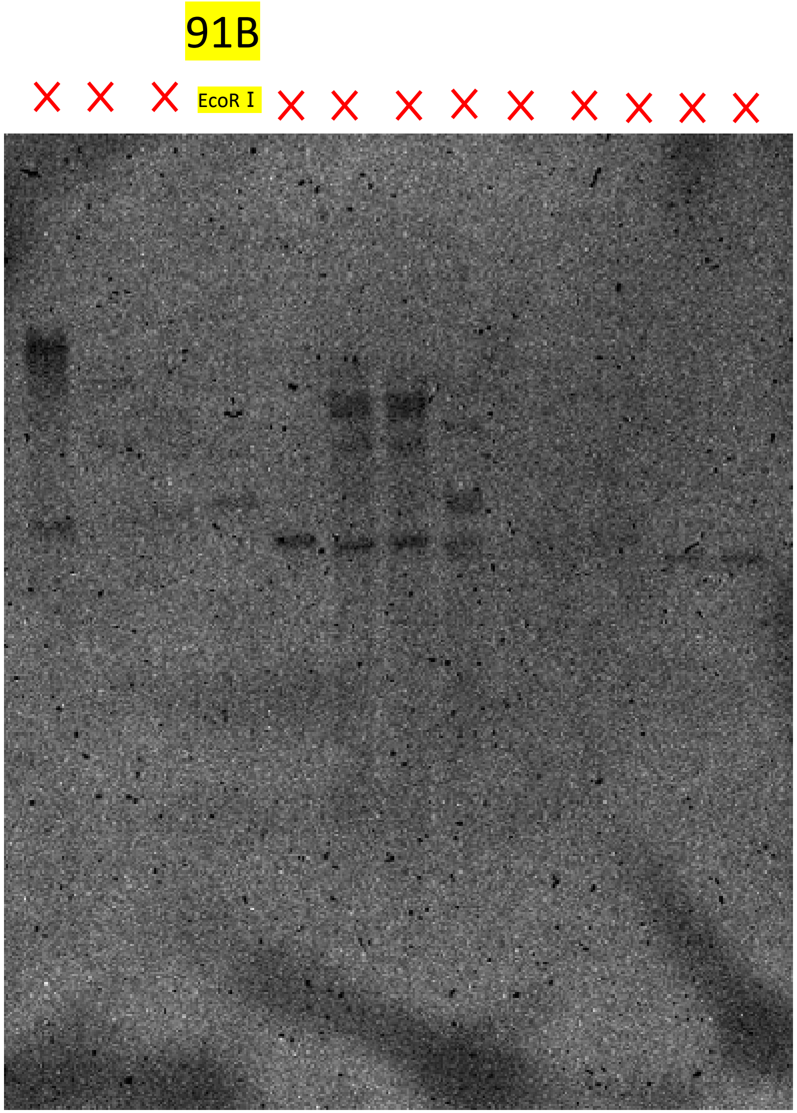

Confirmation of the copy number of stansgene by Southern hybridization

The sample in lane 1 are the same sample as lane 3 in blot images-1.

S4B Fig. blot images-3

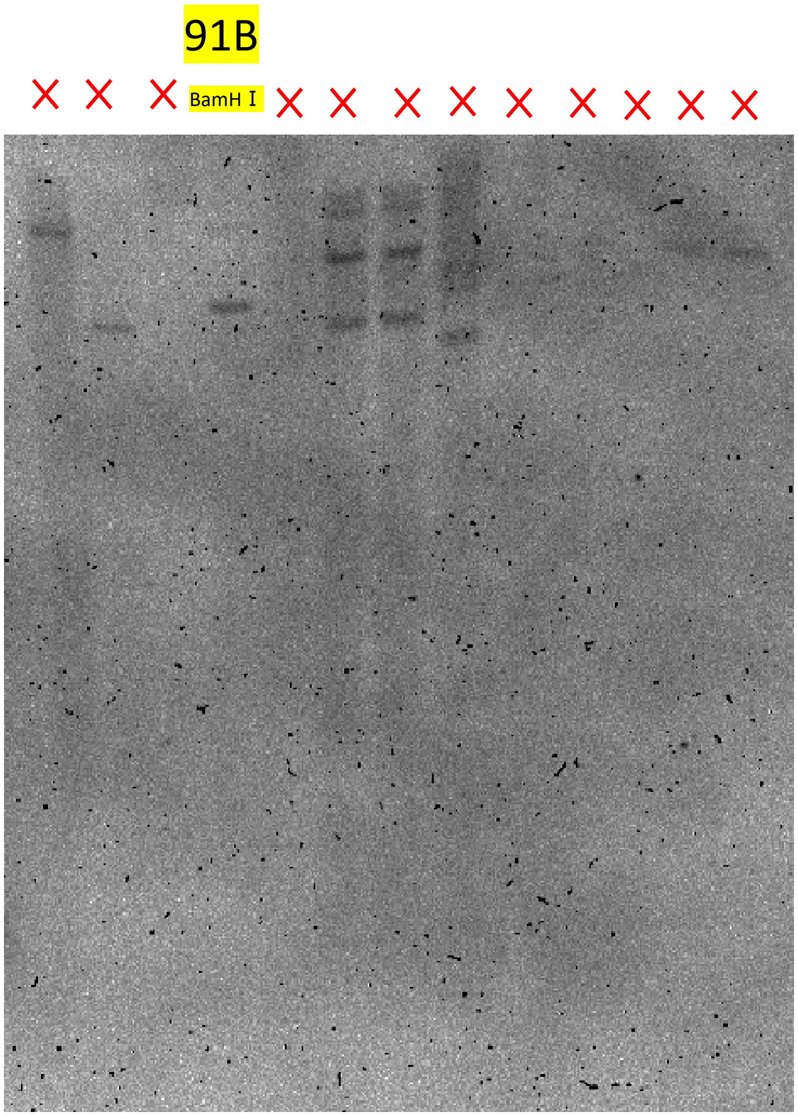

Confirmation of the copy number of stansgene by Southern hybridization

The sample in lane 1 are the same sample as lane 4 in blot images-1.

S7A Fig. gel and blot images

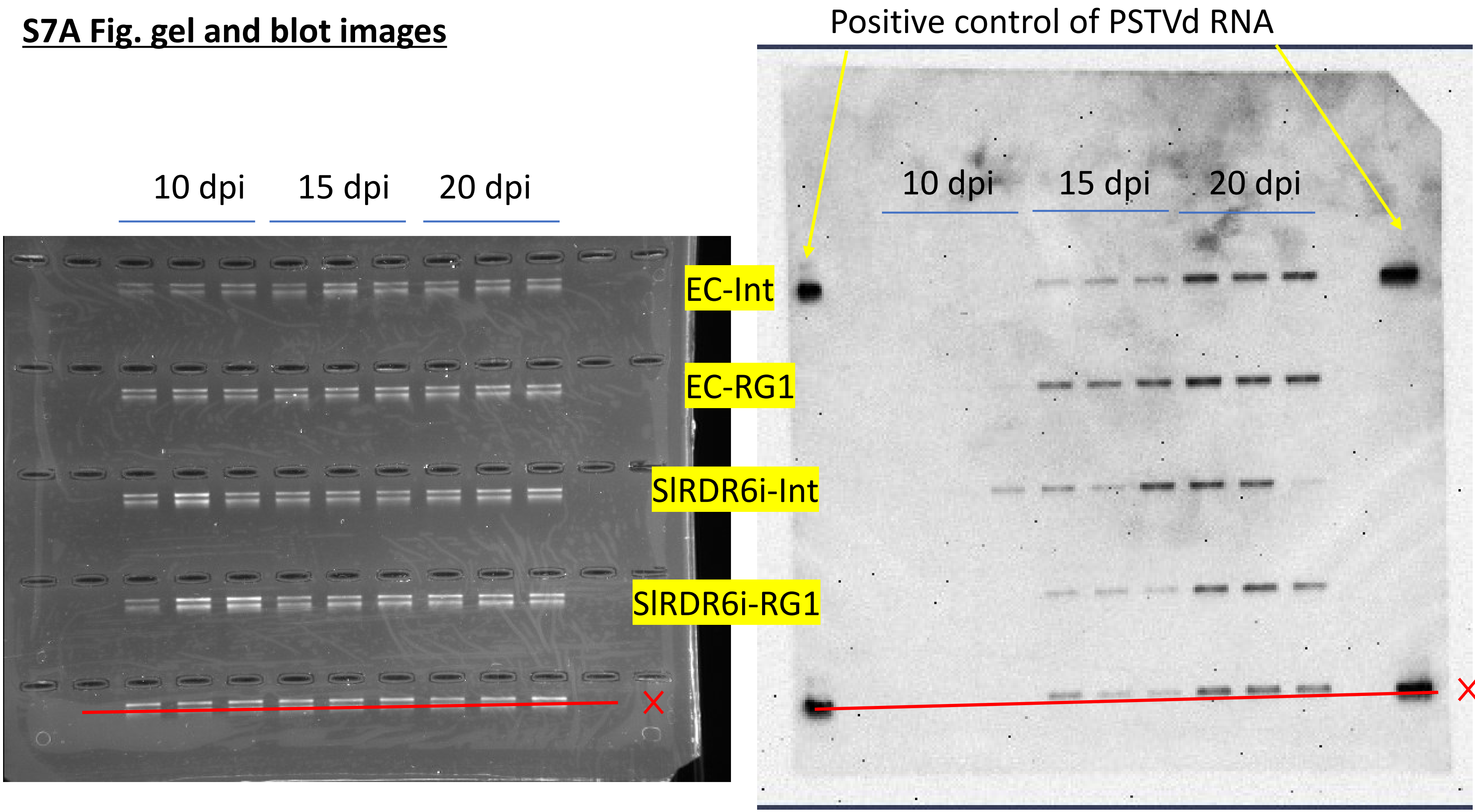

Detection of PSTVd RNA by Northern hybridization

The gel and blot images were taken on separate agarose gel electrophoresis using the same samples.

The lane of the SlRDR6i-Int sample on the blot is shifted to the left by one lane.
